# Analysis of the yeast short-term Crabtree effect and its origin

**DOI:** 10.1111/febs.13019

**Published:** 2014-09-26

**Authors:** Arne Hagman, Torbjörn Säll, Jure Piškur

**Affiliations:** Department of Biology, Lund UniversityLund, Sweden

**Keywords:** aerobic fermentation, Crabtree effect, evolution, *Saccharomyces*, yeast

## Abstract

The short-term Crabtree effect is defined as the immediate occurrence of aerobic alcoholic fermentation in response to provision of a pulse of excess sugar to sugar-limited yeast cultures. Here we have characterized ten yeast species with a clearly defined phylogenetic relationship. Yeast species were cultivated under glucose-limited conditions, and we studied their general carbon metabolism in response to a glucose pulse. We generated an extensive collection of data on glucose and oxygen consumption, and ethanol and carbon dioxide generation. We conclude that the *Pichia*,*Debaryomyces*,*Eremothecium* and *Kluyveromyces marxianus* yeasts do not exhibit any significant ethanol formation, while *Kluyveromyces lactis* behaves as an intermediate yeast, and *Lachancea*,*Torulaspora*,*Vanderwaltozyma* and *Saccharomyces* yeasts exhibit rapid ethanol accumulation. Based on the present data and our previous data relating to the presence of the long-term Crabtree effect in over 40 yeast species, we speculate that the origin of the short-term effect may coincide with the origin of the long-term Crabtree effect in the Saccharomycetales lineage, occurring ∼ 150 million years ago.

## Introduction

One of the most prominent features of baker's yeast (*Saccharomyces cerevisiae*) is the rapid conversion of sugars to ethanol and carbon dioxide under both anaerobic and aerobic conditions. Under aerobic conditions, respiration is possible, with oxygen as the final electron acceptor, but *S. cerevisiae* nonetheless exhibits alcoholic fermentation until the sugar/glucose is depleted from the medium. This phenomenon is called the Crabtree effect 1. However, it is possible to obtain pure respiratory growth under aerobic conditions if the glucose concentration in the medium is kept very low, e.g. by using a glucose-limited continuous culture operating below a certain strain-specific threshold value (called the critical dilution rate) or by using fed-batch cultivation 2. Briefly, the yeast cell senses glucose, and this signal is transmitted further to diminish respiratory activities, but the complexity of glucose repression regulatory networks is still far from being completely understood. Although glucose sensing and the corresponding regulatory mechanisms are relatively well understood in *S. cerevisiae* 3,4, they have been less well studied in other yeast species.

Various physiological and molecular approaches have been used as the background for the current definitions of the Crabtree effect 5–8. In this study, we follow the generally accepted definitions, and describe the long-term Crabtree effect as aerobic alcoholic fermentation under steady-state conditions at high growth rates 8. When *S. cerevisiae* is cultivated in a glucose-limited chemostat, the long-term effect appears when the dilution rate exceeds a strain-specific threshold value. The same effect is also observed when yeast cells are cultivated at high glucose concentration conditions, e.g. batch cultivations 9,10. The underlying mechanism of this physiological trait is not yet fully understood, but it is believed to be caused by the repression of genes involved in respiration 1,13. On the other hand, we define the short-term Crabtree effect as the immediate appearance of aerobic alcoholic fermentation upon addition of excess sugar to sugar-limited and respiratory cultures, as proposed by Pronk *et al*. 11. This effect has also been explained as an overflow in sugar metabolism, initially caused by physiological constraints that may be associated directly with biochemical properties, such as maximum velocity and feedback regulation, of respiration-associated enzymes and their regulators 9,11,12. In addition, it may depend on immediate repression of genes involved in respiration. So far, it is not clear what regulatory mechanisms are shared between the short- and long-term Crabtree effects. A very interesting aspect of this phenomenon, that is still not well understood 13, concerns the origin of the Crabtree effect and its evolutionary background.

Several scenarios for the origin of the long-term Crabtree effect exist. For instance, there may be a single origin of the Crabtree effect after divergence of the *Lachancea*/*Saccharomyces* and *Kluyveromyces*/*Eremothecium* lineages 14. However, isolation and characterization of additional yeast species may reveal other scenarios, i.e. that the Crabtree effect may have appeared before the divergence and been lost by most of *Kluyveromyces*/*Eremothecium* yeasts. It may have appeared gradually (as discussed previously 14) or may have appeared several times independently. Different scenarios may also operate for evolution of the short- and long-term Crabtree effects, i.e. the two processes may have originated as one event or two/several independent events.

Until recently, very few yeast species have been systematically studied with respect to their carbon metabolism 15. Studies on various yeasts with a clear phylogenetic relationship may help to elucidate the evolutionary history of yeast carbon metabolism. In recent work, we studied the presence of the long-term Crabtree effect in over 40 Saccharomycetales yeasts, and it was found that this effect originated after divergence of the *Saccharomyces*/*Lachancea* and *Kluyveromyces*/*Eremothecium* lineages, prior to the whole-genome duplication (WGD) event and after loss of respiratory complex I 14. We felt that it would be interesting to complement these studies and assess the situation regarding the short-term Crabtree effect.

Only a few yeast species have so far been cultivated as continuous cultures and subsequently studied for their glucose metabolism. In this study, we cultured and studied ten yeast species, roughly covering the phylogenetic tree of Saccharomycetales, under sugar-limited conditions. All bioreactor cultivations were performed under the same experimental conditions. We studied the disappearance of glucose and appearance of ethanol, and/or other fermentation and respiration products, in response to glucose pulses. The *Pichia*,*Debaryomyces* and *Eremothecium* yeasts and *Kluyveromyces marxianus* did not exhibit a short-term Crabtree effect. The other tested *Kluyveromyces* species, and the *Lachancea*,*Torulaspora*,*Vanderwaltozyma* and *Saccharomyces* yeasts exhibited rapid ethanol accumulation.

## Results and Discussion

### Continuous cultivation under glucose-limited conditions

In this study, we investigated ten yeast species (Table1), which were selected on the basis of their phylogenetic relationship and their ability to be cultivated in minimal medium under glucose-limited conditions. From the phylogenetic point of view, we have attempted to cover over 200 million years of yeast evolutionary history. Two of the yeasts, *Saccharomyces cerevisiae* and *Vanderwaltozyma polyspora*, belong to the WGD clade, which originated ∼ 100 million years ago. The *Torulaspora*,*Lachancea*,*Kluyveromyces* and *Eremothecium* yeasts separated from the WGD lineage ∼ 100–150 million years ago. The *Debaryomyces* and *Pichia* yeasts used in this study are much more distant relatives, and separated from the other lineages at least 200 million years ago. *S. cerevisiae*,*Lachancea kluyveri*,*Kluyveromyces lactis*,*Kluyveromyces marxianus* and *Pichia pastoris* have previously been cultivated as continuous cultures under fully aerobic conditions 16. However, we report here for the first time that *Eremothecium coryli*,*Debaryomyces vanrijiae*,*Lachanceawaltii*,*Torulaspora franciscae* and *Vanderwaltozymapolysporus* may be successfully cultivated under glucose-limited conditions, and their general carbon metabolism studied (Table2 and Fig. S1).

**Table 1:** Details of the species studied in this paper. Characterized species names, designation and the various collections (Y, CBS and other) from which they may be obtained are provided. All species except *V. polyspora* were investigated in duplicate, and referred to as the species names followed by experiment A and B (as shown in Fig. S1). ‘Y’ indicates the Piskur collection at Lund University, Sweden. ‘CBS’ indicates the Fungal Biodiversity Centre at Utrecht, The Netherlands. The origin of *P. pastoris* has been described previously 14. Yeast species were verified by sequencing of their D1/D2 locus at the end of each experiment. SCP indicates short-term Crabtree-positive (data from this study) and LCP indicates long-term Crabtree positive (data from our previous study 14).

Species	Y	CBS	Other	D1/D2accession number	SCP	LCP
*S. cerevisiae* A/B	Y706	8340	CEN.PK 113-7D	HM107792.1	+++	+++
*V. polysporus*	Y1293	2163	NRRL Y-8283	AY048169.1	+++	+++
*T. franciscae* A/B	Y1055	2926	NRRL Y-6686	JF781360.1	++	++
*L. waltii* A/B	Y1062	6430	NRRL Y-8285	U69582.1	++	++
*L. kluyverii* A/B	Y1651		UWOPS79-150	GU213454.1	++	++
*K. lactis* A/B	Y707	2359	NRRL Y-1140	GQ121694.1	+	−
*K. marxianus* A/B	Y1058	712	NRRL Y-8281	JN938927.1	−	−
*E. coryli* A/B	Y999	2608	NRRL Y-12970	U43390.1	−	−
*D. vanrijiae* A/B	Y060	3024	NRRL Y-7430	AB281295.1	−	−
*P. pastoris* A/B	Y1294			FR839631.1	−	−

**Table 2:** Yeast short-term Crabtree effect: growth parameters at early time points.

Species	Time	Glucose (g·L^−1^)	Ethanol (g·L^−1^)	DW (g·L^−1^)	O_2_ (mmol g DW^−1^·h^−1^)	CO_2_ (mmol g DW^−1^·h^−1^)	RQ
*S. cerevisiae* A/B	SS	0.0 ± 0.0	0.00 ± 0.01	3.7 ± 0.2	2.7 ± 0.1	2.7 ± 0.09	1.0 ± 0.0
	5	9.1 ± 0.1	0.04 ± 0.00	3.6 ± 0.1	2.6 ± 0.1	4.1 ± 0.03	1.6 ± 0.4
	20	8.5 ± 0.0	0.17 ± 0.00	3.7 ± 0.1	3.8 ± 0.1	7.0 ± 0.30	1.8 ± 0.0
	40	7.8 ± 0.2	0.36 ± 0.03	3.8 ± 0.1	3.7 ± 0.5	7.4 ± 0.62	2.0 ± 0.1
	60	6.5 ± 0.1	0.63 ± 0.11	4.1 ± 0.2	3.8 ± 0.4	7.8 ± 0.07	2.1 ± 0.0
*V. polysporus*	SS	0.0	0.00	3.1	2.7	2.8	1.0
	10	6.8	0.19	2.4	3.8	5.2	1.3
	20	6.2	0.26	2.5	4.2	8.9	2.1
	40	5.4	0.60	2.7	3.7	9.8	2.6
	60	4.3	0.93	2.8	3.7	10.0	2.7
*T. franciscae* A/B	SS	0.0 ± 0.0	0.00 ± 0.00	4.2 ± 0.2	1.3 ± 0.2	2.2 ± 0.1	1.7 ± 0.3
	5	9.2 ± 0.1	0.01 ± 0.01	4.1 ± 0.2	1.3 ± 0.5	2.1 ± 0.1	1.7 ± 0.7
	20	8.8 ± 0.0	0.08 ± 0.00	4.3 ± 0.1	2.4 ± 0.1	4.4 ± 0.2	1.8 ± 0.0
	40	8.3 ± 0.0	0.18 ± 0.02	4.4 ± 0.2	2.4 ± 0.2	4.8 ± 0.2	2.0 ± 0.1
	60	7.7 ± 0.0	0.31 ± 0.01	4.4 ± 0.1	2.6 ± 0.1	5.5 ± 0.2	2.1 ± 0.0
*L. waltii* A/B	SS	0.0 ± 0.0	0.00 ± 0.00	4.2 ± 0.1	3.0	2.6	0.9
	5	9.3 ± 0.4	0.01 ± 0.01	4.4 ± 0.2	3.7	2.5	0.7
	20	8.6 ± 0.3	0.14 ± 0.01	4.8 ± 0.1	3.5	5.5	1.6
	40	7.7 ± 0.3	0.29 ± 0.02	4.8 ± 0.2	3.3	6.1	1.9
	60	6.7 ± 0.3	0.47 ± 0.00	5.0 ± 0.0	3.2	6.0	1.9
*L. kluyverii* A/B	SS	0.1 ± 0.0	0.01 ± 0.00	3.6 ± 0.1	3.1 ± 0.2	3.1 ± 0.2	1.0 ± 0.0
	5	8.7 ± 0.1	0.01 ± 0.01	3.5 ± 0.0	3.3 ± 0.2	3.2 ± 0.3	1.0 ± 0.0
	20	8.2 ± 0.0	0.07 ± 0.02	3.4 ± 0.1	4.2 ± 0.3	5.6 ± 0.0	1.3 ± 0.1
	40	7.7 ± 0.1	0.25 ± 0.00	3.4 ± 0.0	4.4 ± 0.3	6.9 ± 0.2	1.6 ± 0.1
	60	7.1 ± 0.1	0.48 ± 0.07	3.6 ± 0.1	4.3 ± 0.4	7.6 ± 0.3	1.8 ± 0.1
*K. lactis* A/B	SS	0.0 ± 0.0	0.00 ± 0.00	3.6 ± 0.0	2.2 ± 0.1	2.7 ± 0.3	1.3 ± 0.1
	5	9.4 ± 0.0	0.01 ± 0.01	3.6 ± 0.2	2.7 ± 0.0	4.2 ± 0.1	1.5 ± 0.1
	20	8.9 ± 0.1	0.03 ± 0.03	3.8 ± 0.2	4.5 ± 0.1	5.0 ± 0.3	1.1 ± 0.0
	40	8.2 ± 0.0	0.09 ± 0.04	4.1 ± 0.1	4.2 ± 0.1	5.3 ± 0.3	1.3 ± 0.1
	60	7.4 ± 0.0	0.22 ± 0.01	4.3 ± 0.2	4.3 ± 0.1	5.5 ± 0.4	1.3 ± 0.1
*K. marxianus* A/B	SS	0.0 ± 0.0	0.00 ± 0.00	4.0 ± 0.0	0.9 ± 0.1	0.9 ± 0.0	1.0 ± 0.0
	5	9.5 ± 0.0	0.00 ± 0.01	3.9 ± 0.0	1.2 ± 0.1	1.0 ± 0.0	0.9 ± 0.0
	20	8.9 ± 0.1	0.00 ± 0.00	4.2 ± 0.0	1.5 ± 0.1	1.5 ± 0.0	1.0 ± 0.0
	40	8.5 ± 0.1	0.00 ± 0.00	4.5 ± 0.0	1.5 ± 0.0	1.5 ± 0.0	1.0 ± 0.0
	60	7.9 ± 0.1	0.00 ± 0.00	4.8 ± 0.0	1.5 ± 0.1	1.5 ± 0.0	1.0 ± 0.0
*E. coryli* A/B	SS	0.0 ± 0.0	0.00 ± 0.00	5.8 ± 0.3	0.8 ± 0.0	0.7 ± 0.0	0.9 ± 0.0
	5	9.8 ± 0.0	0.00 ± 0.00	5.6 ± 0.2	0.8 ± 0.0	0.8 ± 0.0	1.0 ± 0.1
	20	9.7 ± 0.1	0.02 ± 0.01	5.8 ± 0.2	0.7 ± 0.0	0.8 ± 0.1	1.1 ± 0.0
	40	9.4 ± 0.2	0.02 ± 0.02	5.9 ± 0.2	0.7 ± 0.1	0.8 ± 0.1	1.1 ± 0.0
	60	9.2 ± 0.2	0.06 ± 0.04	6.0 ± 0.3	0.6 ± 0.1	0.8 ± 0.0	1.2 ± 0.1
*D. vanrijiae* A/B	SS	0.0 ± 0.0	0.00 ± 0.00	4.4 ± 0.0	2.0 ± 0.0	1.8 ± 0.1	0.9 ± 0.1
	5	9.5 ± 0.2	0.01 ± 0.01	4.2 ± 0.0	2.2 ± 0.0	1.8 ± 0.3	0.8 ± 0.1
	25	9.2 ± 0.1	0.00 ± 0.00	4.3 ± 0.0	2.3 ± 0.0	2.3 ± 0.1	1.0 ± 0.0
	50	8.9 ± 0.1	0.00 ± 0.00	4.6 ± 0.0	1.8 ± 0.0	2.1 ± 0.1	1.1 ± 0.0
	70	8.6 ± 0.1	0.00 ± 0.00	4.9 ± 0.1	1.7 ± 0.1	2.0 ± 0.1	1.2 ± 0.1
*P. pastoris* A/B	SS	0.0 ± 0.0	0.00 ± 0.00	4.8 ± 0.1	1.3 ± 0.0	1.5 ± 0.1	1.1 ± 0.1
	5	8.1 ± 0.7	0.00 ± 0.00	4.7 ± 0.1	1.7 ± 0.0	1.7 ± 0.0	1.0 ± 0.0
	20	7.5 ± 0.6	0.00 ± 0.00	5.2 ± 0.2	2.1 ± 0.1	2.4 ± 0.0	1.2 ± 0.0
	40	7.0 ± 0.6	0.00 ± 0.00	5.5 ± 0.3	1.9 ± 0.0	2.3 ± 0.0	1.2 ± 0.0
	80	6.0 ± 0.7	0.00 ± 0.00	6.4 ± 0.1	1.6 ± 0.1	1.9 ± 0.1	1.2 ± 0.0

Values for each growth parameter are means ± standard deviation. SS, steady state.

Using bioreactors, we prepared steady-state yeast cultures that were fully respiring and non-oscillating, and glucose was the only carbon source and the growth-limiting factor. The culture was then pulsed with glucose to a final concentration of 8–10 g·L^−1^ (time point 0). We then followed the consumption of glucose and O_2_, and the increase in biomass and CO_2_ and appearance of any glucose catabolic product, such as ethanol (Fig. S1). An overview of these results is provided in Table2 and Table S1, and the results are thoroughly discussed in the subsequent sections.

### Consumption of glucose and generation of biomass and ethanol

Yeast growth was monitored by following changes in the dry weight (DW) and attenuance at 600 nm at various time points. The results, summarized in Table2, showed that growth was unchanged in all tested yeasts during the first hour. In other words, no sudden conversion of glucose into additional biomass was observed. Growth stabilized for the various yeasts at time points later than 60 min (Table S1).

The *Pichia*,*Debaryomyces*,*Eremothecium* and *K. marxianus* yeasts did not exhibit any significant ethanol formation after a glucose pulse. All the other tested members of the *Kluyveromyces*,*Lachancea* and *Torulaspora* clades, which belong to pre-WGD lineages, exhibited a relatively weak ethanol accumulation in the range 0.22–0.48 g·L^−1^ during the first hour, while the *Saccharomyces* and *Vanderwaltozyma* yeasts exhibited rapid and high ethanol accumulation in the range 0.63–0.93 g·L^−1^ (Table2). Ethanol accumulation in response to a glucose pulse was also monitored in all yeast species. All fermenting yeasts exhibited significant ethanol formation within the first 20 min in response to a glucose pulse (see also Fig. S2). All fermenting yeast species are hereafter referred to as short-term Crabtree positive (SCP) yeasts, and all non-fermenting yeast species are referred to as short-term Crabtree negative (SCN) yeasts (Table1).

**Figure 1:**
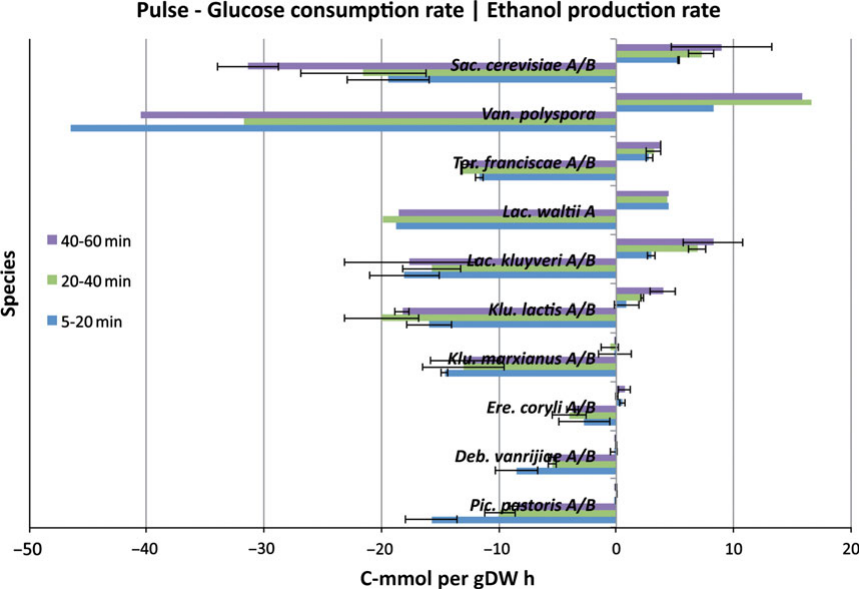
High glucose consumption rates indicate a high glycolytic flux, which is necessary for high flux through fermentative pathways that result in ethanol production. It has previously been shown that Crabtree-positive yeast species show high glucose consumption rates 14. Here we show that the glucose uptake rates and ethanol production rates for all fermenting yeasts are equal to or higher than *K. marxianus*, which is the SCN yeast with the highest initial glucose uptake rate in our experiments. Error bars correspond to the standard deviation for the mean glucose uptake rates (left) and ethanol production rates (right) between biological replicates. Values are means at time points 5–20 min (blue), 20–40 min (green) and 40–60 min (purple) after a glucose pulse for all species, except *P. pastoris* and *D. vanrijiae*. The corresponding time points for *P. pastoris* are 5–20, 20–40 and 40–80 min, and those for *D. vanrijiae* are 5–25, 25–50 and 50–70 min. The yeast species are organized according to their phylogenetic position, with *S. cerevisiae* at the top, followed by a decreasing phylogenetic relationship with the most distant related yeast species below, in the same order as previously reported 14.

We also monitored glucose consumption by determining the glucose concentration at various time points for several yeasts. The results summarized in Table2 show that all yeasts continued to consume glucose after a glucose pulse (see also Fig. S3). However, the initial glucose consumption rates are unstable, and no clear pattern between rates and phylogenetic position was determined (Fig.1). However, a general distinction between SCN and SCP yeasts was observed based on glucose consumption rates. All SCN yeasts had a mean initial glucose consumption rate in the range 2.7–15.8 C-mmol g DW^−1^·h^−1^, which was significantly different (*P* value = 9.1 × 10^−3^ Table3) from that for the SCP yeasts (11.7–46.4 C-mmol g DW^−1^·h^−1^) (Table S1).

**Table 3:** Results of statistical tests amongst two metabolic groups of yeasts with respect to investigated growth parameters. The test results are considered significant at a significance level of α = 5%. Yeast species were grouped in the following way. The first group comprises SCN yeasts (but not *K. lactis*), and the second group comprises all SCP yeasts (including *K. lactis*). Performing pairwise parametric and non-parametric tests on SCN and SCP yeasts revealed significant differences between groups for all investigated parameters. In other words, SCN and SCP yeasts may be grouped according to their glucose and O_2_ consumption rates, ethanol and CO_2_ production rates, and RQ value. DF stands for degree of freedom.

Statistical analysis	*P* value	test statistic	DF
Welch *t* test: glucose consumption rate	1.7E-02	2.7	16.6
Welch *t* test: ethanol production rate	1.7E-04	5.4	12.1
Welch *t* test: O_2_ consumption rate	8.8E-05	5.2	16.0
Welch *t* test: CO_2_ production rate	3.5E-06	7.5	13.7
Welch *t* test: RQ	8.4E-04	4.6	10.4
Mann–Whitney: glucose consumption rate	9.1E-03	13	–
Mann–Whitney: ethanol production rate	8.2E-04	3	–
Mann–Whitney: O_2_ consumption rate	2.9E-03	6	–
Mann–Whitney: CO_2_ production rate	4.6E-05	0	–
Mann–Whitney: RQ	4.5E-04	0	–

### Consumption of O_2_, production of CO_2_, and RQ

**Figure 2:**
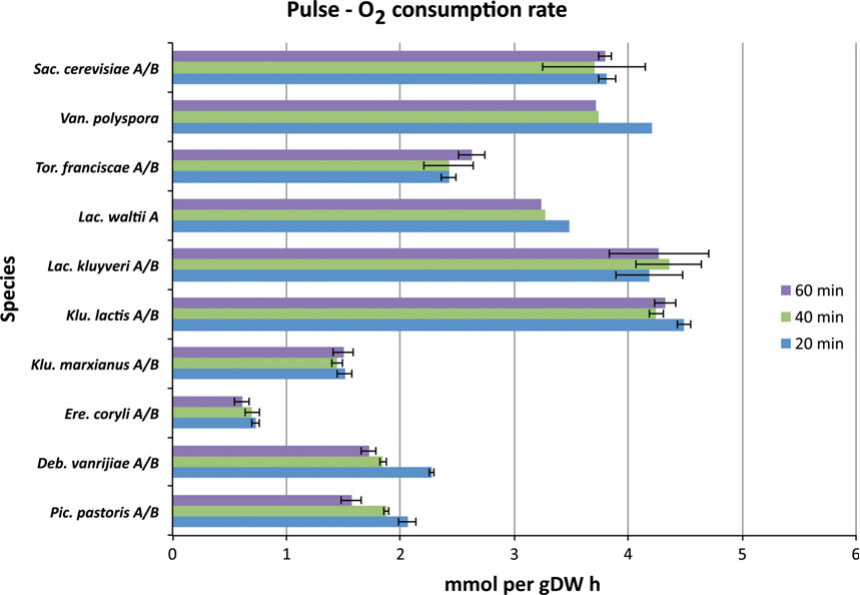
Oxygen consumption rates for various yeast species. It is known from batch experiments that repression of respiration may contribute to increased ethanol production rates, but no such effect was observed within the first hour for any short-term Crabtree-positive yeasts. Values are mean oxygen consumption rates for biological replicates at the time points 20 min (blue), 40 min (green) and 60 min (purple) for all species, except *P. pastoris* and *D. vanrijiae*. The corresponding time points for *P. pastoris* are 20, 40 and 80 min, and those for *D. vanrijiae* are 25, 50 and 70 min (see also Fig. S4 for additional time points). Error bars correspond to the standard deviation. The yeast species are organized according to their phylogenetic position, with *S. cerevisiae* at the top, followed by a decreasing phylogenetic relationship in the same order as previously reported 14.

When respiring yeasts proliferate on glucose, O_2_ is consumed and the respiratory activity may be quantified from the O_2_ consumption. Our data show that SCN yeasts had an initially lower respiration compared to SCP yeasts in response to a glucose pulse (Fig.2 and Fig. S4). All SCN yeasts had an initial O_2_ consumption in the range 0.8–2.2 mmol g DW^−1^·h^−1^, which was significantly different (*P* value = 2.9 × 10^−3^, Table3) from that of the SCP yeasts (1.9–4.0 mmol g DW^−1^·h^−1^) (Table S2).

**Figure 3:**
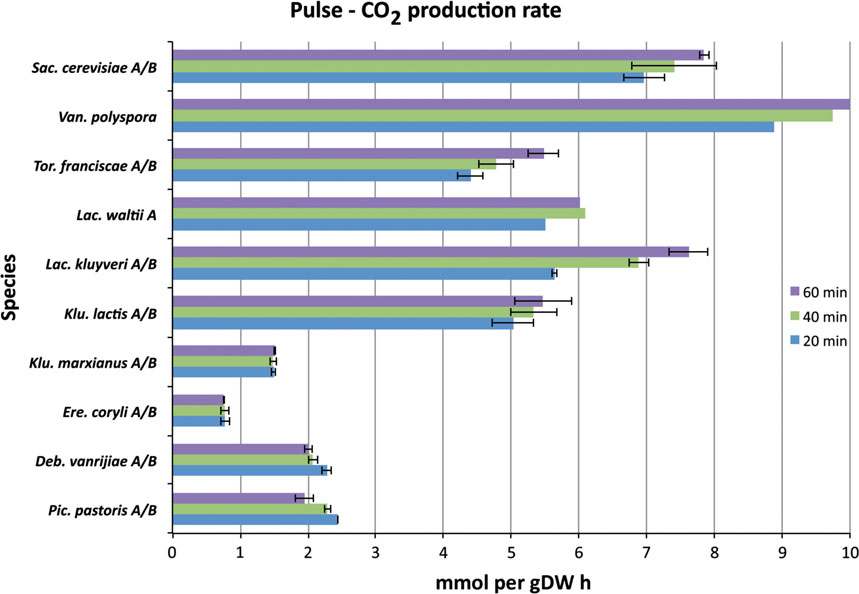
Carbon dioxide production rates for various yeast species. Carbon dioxide is produced from both respiration and fermentation, and high carbon dioxide production rates are often associated with fermentative activity in Crabtree-positive yeasts such as *S. cerevisiae* (explained in Fig.4). A significant increase in the carbon dioxide production rate was observed after 40 min in Crabtree-positive yeasts such as *S. cerevisiae*,*T. franciscae* and *L. kluyverii*, but no such effect was observed within the first hour for any non-fermenting yeast. Values are mean carbon dioxide production rates between biological replicates, at time points 20 min (blue), 40 min (green) and 60 min (purple) after a glucose pulse for all species, except *P. pastoris* and *D. vanrijiae*. The corresponding time points for *P. pastoris* are 20, 40 and 80 min, and those for *D. vanrijiae* are 25, 50 and 70 min (see also Fig. S5 for additional time points). Error bars correspond to the standard deviation. The yeast species are organized according to their phylogenetic position, with *S. cerevisiae* at the top, followed by a decreasing phylogenetic relationship in the same order as previously reported 14.

CO_2_ is primarily produced from respiration and fermentation, and, when we compared the rates of CO_2_ production between yeasts, SCN yeasts exhibited initially lower CO_2_ production rates compared to SCP yeasts in response to a glucose pulse (Fig.3 and Fig. S5). The initial CO_2_ production rates for SCN yeast were in the range 0.8–2.1 mmol g DW^−1^·h^−1^, which was significantly different (*P* value = 4.6 × 10^−5^, Table3) from that for the SCP yeasts (3.2–7.0 mmol g DW^−1^·h^−1^) (Table S2).

**Figure 4:**
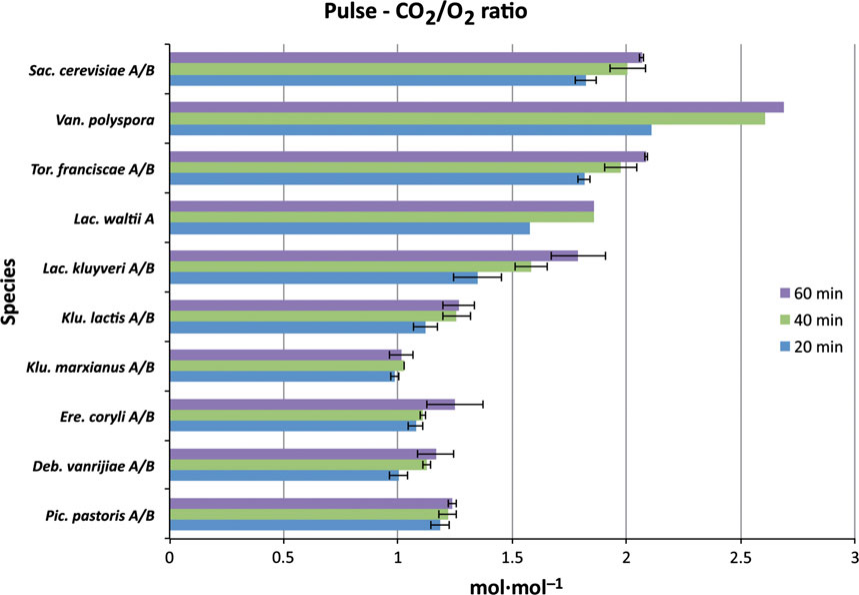
Under purely respiratory metabolism with low biomass formation, roughly one carbon dioxide molecule is produced for each consumed oxygen molecule. Fermentation does not require oxygen as the final electron acceptor, and only produces carbon dioxide in the first decarboxylation step of pyruvate. Hence, RQ values > 1 indicate fermentative activity. All ethanol-forming yeasts have RQ values significantly > 1, while all non-ethanol forming yeasts have an RQ close to or equal to 1. As no significant decrease in oxygen consumption rates was observed in fermenting yeasts during the first hour after a glucose pulse (see also Fig.2), the increase in RQ observed after 40 min for fermenting yeasts may only be caused by up-regulation of fermentative pathways, as indicated by increased carbon dioxide rates (see also Fig.2). Values are mean carbon dioxide production rates between biological replicates at time points 20 min (blue), 40 min (green) and 60 min (purple) after a glucose pulse for all species, except *P. pastoris* and *D. vanrijiae*. The corresponding time points for *P. pastoris* are 20, 40 and 80 min, and those for *D. vanrijiae* are 25, 50 and 70 min (see also Fig. S6 for additional time points). Error bars correspond to the standard deviation.

The inter-relationship between fermentative and respiratory activity may be deduced from the respiratory quotient (RQ), which is the ratio between produced CO_2_ and consumed O_2_. A RQ value of 1 indicates a fully aerobic metabolism, and values > 1 are consistent with fermentative metabolism. As for glucose and O_2_ consumption, and ethanol and CO_2_ production, a significant difference in RQ values (*P* value = 8.4 × 10^−4^, Table3) was observed between SCP and SCN yeasts (Fig.4 and Fig. S6). SCN yeasts had a RQ value close to 1 (range of 1.0–1.1), while all SCP yeasts had higher RQ values (range 1.2–2.4) (Table S2).

### Comparison of the short-term and long-term Crabtree effects

When we compare the phylogenetic distribution of the short-term and long-term Crabtree effects in all investigated yeast species, the results are congruent with the exception of *K. lactis* (Table1 and Fig. S7). We show that *S. cerevisiae* and *V. polyspora* are both strong short- and long-term Crabtree-positive, *T. franciscae*,*L. waltii* and *L. kluyverii* are both moderately short- and long-term Crabtree-positive, *K. marxianus*,*E. coryli*,*D. vanrijiae* and *P. pastoris* are both short- and long-term Crabtree-negative, and *K. lactis* is weak short-term Crabtree-positive but long-term Crabtree-negative.

It is clear that WGD yeast species show initially higher fermentative activity after a glucose pulse compared to pre-WGD yeasts, with *K. lactis* exhibiting the lowest fermentative activity among all the fermenting yeasts. Furthermore, increasing initial fermentative activity that reflects the phylogenetic position was observed among yeasts as deduced from the RQ values (Fig.4). The above results regarding CO_2_ production rates, O_2_ consumption rates and the ratio of these are consistent with our data on ethanol production rates and glucose consumption rates. Although more variable than the RQ values, it is clear that the glucose consumption rates are similar to, or higher, in all the SCP yeasts compared to non-fermenting yeasts (Fig.1). It may also be concluded that *K. lactis* exhibits the weakest short-term Crabtree effect, reflecting its intermediate phylogenetic position between SCN and SCP yeasts. The weak short-term Crabtree effect in *K. lactis* is manifested as initially high CO_2_ production (Fig.3 and Fig. S5) and O_2_ consumption rates (Fig.2 and Fig. S4) that are significantly different from those of SCN yeasts (*P* value = 6.4 × 10^−3^, Table S4) but not significantly different from those of other SCP yeasts (*P* value = 7.0 × 10^−1^, Table S4). However, the low RQ value (Fig.4) and low ethanol production and glucose consumption rates (Fig.1) were not significantly different from those of SCN yeasts (*P* values > 3.2 × 10^−1^, Table S4). Furthermore, we did not observe any relationship between long- and short-term Crabtree effects and oxygen consumption rates.

The results of our study suggest that *Kluyveromyces* yeasts may be considered as intermediate in terms of both their phylogenetic position and their carbon metabolism. *K. lactis* is short-term Crabtree-positive (present results), but long-term Crabtree-negative (Table1 and Fig. S7) 14. Other investigated yeast species that diverged from the rest of the *Saccharomyces* lineage prior to, or at approximately the same time as *K. lactis*, such as *P. pastoris*,*D. vanrijiae*,*E. coryli* and *K. marxianus*, are both short- and long-term Crabtree-negative 14. Although the number of studied yeasts in our paper is only ten, we speculate that the time of origin of the short-term Crabtree effect and the time of origin of the long-term Crabtree effect are very close to each other and may even overlap, coinciding with the horizontal transfer of *URA1* gene (encoding a bacterial ‘anaerobic’ DHODase) and the ability to proliferate anaerobically (Fig. S7) 14,17. Furthermore, this study has generated a large collection of data that may be used to develop a model to elucidate the mechanisms behind the observed results and help to reconstruct the evolutionary pathways leading to the modern traits in extant yeasts.

## Experimental procedures

### Yeast strains

In this study, we investigated ten yeast species, roughly covering the Saccharomycetales, for their rapid response to glucose under aerobic and glucose-limited conditions: *Saccharomyces cerevisiae* Y706, *Vanderwaltozyma polysporus* Y1293, *Torulaspora franciscae* Y1055, *Lachancea waltii* Y1062, *Lachancea kluyverii* Y1651, *Kluyveromyces lactis* Y707, *Kluyveromyces marxianus* Y1058, *Eremothecium coryli* Y999, *Debaryomyces vanrijiae* Y060 and *Pichia pastoris* Y1294 (Table1). The identity of each species was verified by sequencing of the final culture at the end of each experiment (Table1) as previously described 14.

### Steady-state conditions and glucose pulse

All cultivations were performed in duplicate (except for *V. polyspora*) in Multifors bioreactors (INFORS HT, Bottmingen/Basel, Switzerland), to ensure controlled and uniform growth conditions. The temperature was maintained at 25 °C, the pH was maintained at 5 (± 0.5 units), the airflow was maintained at 0.7 L*min^−1^, and the stirrer speed was varying from 200 to 1200 rpm to ensure a dissolved oxygen concentration above 30% of air saturation. The pH was measured using 405-DPAS-SC-K8S/225 sensors (Mettler Toledo, Greifensee, Switzerland), and the dissolved oxygen was measured using InPro 6800S sensors (Mettler Toledo), as previously described 14. The starting conditions in all experiments comprised fully respiring and non-oscillating steady-state cultures at a constant working volume of 0.7 L, and with growth rates of 0.1 h^−1^. The medium feed contained 40 mm glucose, which was the only limiting carbon source in the defined synthetic medium, prepared as previously reported 18. The growth conditions were kept constant throughout the duration of each experiment except the medium feed, which was turned off prior to a glucose pulse. Steady-state cultures were pulsed with glucose to a final concentration of 8–10 g·L^−1^ (measured at the first time point after a glucose pulse, see Table2). All starting-cultures were grown overnight, and washed before inoculation, in the same minimal medium as used in the bioreactors. Furthermore, 2.0 g·L^−1^ Kaiser synthetic complete supplement (ForMedium, Hunstanton, UK) was added to the medium feed for cultivation of *Eremothecium coryli*, due to its auxotrophic nature.

### Yeast central carbon metabolism

The concentrations of glucose, ethanol, acetate, lactate, succinate and glycerol in the culture supernatant were determined by HPLC 1200 series (Agilent, Santa Clara, CA) as previously reported 14. The various compounds were separated at 60 °C using an Aminex HPX-87H column (BioRad, Hercules, CA, USA), and detected using an refractive index detector set to 55 °C, and a UV detector. The mobile phase consisted of 5 mm H_2_SO_4_, and the flow rate was 0.6 mL·min^−1^. Growth rates were determined based on measurements of the dry weight (DW) and attenuance at 600 nm (Fig. S1), but only the DW was used for calculation of the consumption and production rates of various compounds (Tables S1 and S2). DW was determined as previously reported 14, by weighing GF/A glass microfiber filters (Whatman, Amersham, UK) before and after filtering of washed culture samples that had been dried for 1 day (at 70 °C). Furthermore, growth kinetics were determined only during the glucose consumption phase, before any accumulated ethanol was consumed.

The consumption rates of glucose and the production rates of products (biomass and ethanol) are reported in C-mmol g DW^−1^·h^−1^ (C-mmol glucose or product per gram biomass per hour). For all short-term Crabtree-positive yeast species (except *K. lactis*), which exhibit highly variable growth during the first hour after a glucose pulse, the rates were calculated by taking the amount of consumed glucose or formed product and dividing it by the mean DW and the duration between two time points. The consumption and production rates were calculated differently for time points after the first hour (when growth was stabilized) for short-term Crabtree positive yeasts and for all short-term Crabtree negative yeast species (including *K. lactis*), which immediately start to grow with an invariable growth rate after a glucose pulse. These rates were calculated by taking the amount of consumed glucose or formed product and dividing it by the amount of DW formed, and multiplying by the corresponding growth rate between two time points.

The consumption rates of O_2_ and production rates of CO_2_ were calculated as previously reported 19. These rates were calculated based on the production of CO_2_ and consumption of O_2_, as determined using BC-CO_2_ and BCP-O_2_ gas analyzers (BlueSens, Herten, Germany) as previously described 14. Respiratory quotients (RQ) were calculated by dividing the rate of produced CO_2_ by the rate of consumed O_2_ (Fig. S1 and Table S2).

### Statistical analysis

Statistical analyses were performed using r version 2.15.2 (http://www.cran.r-project.org). Parametric and non-parametric pairwise tests (Welch two-sample *t* test and Wilcoxon rank sum test) on SCN yeasts and SCP yeasts (including *K. lactis*) were used to test for equality of group means of glucose and O_2_ consumption rates, ethanol and CO_2_ production rates, and the RQ (Table3). Two tests for homoscedasticity (Bartlett's *K*^2^ and Levene's test) were then performed on investigated groups and parameters (Table S3). In the main text, *P* values from Welch two sample *t* test are reported for parameters that pass the homoscedasticity test, and *P* values from Wilcoxon rank sum test are reported for parameters fail the homoscedasticity test. To investigate for any bias due to missing data or unequal repetition of experiments among species (see Table S2), similar tests (Welch two-sample *t* test and Wilcoxon rank sum test) were performed on the mean of experimental replicates for each species. We also performed these tests to allow for between replicate correlation. These results (not shown) are consistent with the results in Table3. Thus, we come to the same conclusions, irrespective of methods. All parameters were further investigated using ANOVA, Kruskal–Wallis and Tukey–Kramer tests to assess any significant difference amongst three groups that contain SCN, only *K. lactis*, and SCP (not including *K. lactis*) (Table S4).

## Abbreviations

C-mmol: mmol carbon
DW: dry weight
RQ: respiratory quotient
SCN: short-term Crabtree-negative
SCP: short-term Crabtree-positive
WGD: whole-genome duplication

## Supporting Information

Fig S1 Yeast growth profiles. **Fig. S2.** Ethanol formation in response to a glucose pulse. **Fig. S3.** Continuous glucose consumption in response to a glucose pulse. **Fig. S4.** Oxygen consumption rates (first 100 min). **Fig. S5.** Carbon dioxide production rates (first 100 min). **Fig. S6.** Respiratory quotient (first 100 min). **Fig. S7.** Yeast carbon metabolism and phylogeny. **Table S1.** Yeast short-term Crabtree effect: ethanol, glucose and DW for all time points. **Table S2.** Yeast short-term Crabtree effect: O_2_, CO_2_ and RQ for all time points. **Table S3.** Homogeneity test of variance between two metabolic groups. **Table S4.** Statistical tests among three metabolic groups.
