# Supplementary material for: Analysis of the yeast short-term Crabtree effect and its origin
Source: FEBS J. 2014 Sep 26;281(21):4805–14. doi: 10.1111/febs.13019 (PMC4240471; doi:10.1111/febs.13019)
Supplement: Fig S1 — Yeast growth profiles. Fig. S2. Ethanol formation in response to a glucose pulse. Fig. S3. Continuous glucose consumption in response to a glucose pulse. Fig. S4. Oxygen consumption rates (first 100 min). Fig. S5. Carbon dioxide production rates (first 100 min). Fig. S6. Respiratory quotient (first 100 min). Fig. S7. Yeast carbon metabolism and phylogeny. Table S1. Yeast short-term Crabtree effect: ethanol, glucose and DW for all time points. Table S2. Yeast short-term Crabtree effect: O2, CO2 and RQ for all time points. Table S3. Homogeneity test of variance between two metabolic groups. Table S4. Statistical tests among three metabolic groups. [file febs0281-4805-SD1.pdf]

the FEBS Journal
www.febsjournal.org

# Analysis of the yeast short-term Crabtree effect and its origin

Arne Hagman, Torbjörn Säll and Jure Piškur

DOI: 10.1111/febs.13019

# SUPPORTING INFORMATION

## Analysis on yeast short-term Crabtree effect and its origin

Arne Hagman, Torbjörn Säll and Jure Piškur

*Department of Biology, Lund University, Lund, Sweden*

**Correspondence**

Arne Hagman, Department of Biology, Lund University, Sölvegatan 35, 223 62 Lund, Sweden

Tel: +46 46 22 21 445

E-mail: arne.hagman@biol.lu.se

**List of content**

| | |
|---|---|
| Figure S1: Yeast growth profiles. | p 1-10 |
| Figure S2: Ethanol formation in response to a glucose-pulse. | p 11-12 |
| Figure S3: Continuous glucose consumption in response to a glucose-pulse. | p 13 |
| Figure S4: Oxygen consumption rates (first 100 min). | p 14 |
| Figure S5: Carbon dioxide production rates (first 100 min). | p 15 |
| Figure S6: Respiratory quotient (first 100 min). | p 16 |
| Figure S7: Yeast carbon metabolism and phylogeny. | p 17 |
| Table S1: Yeast short-term Crabtree effect – ethanol, elucose and DW for all time points. | p 18-20 |
| Table S2: Yeast short-term Crabtree effect – $O_2$, $CO_2$ and RQ for all time points. | p 21-23 |
| Table S3: Homogeneity test of variance between two metabolic groups. | p 24 |
| Table S4: Statistical tests among three metabolic groups. | p 25 |
| References | p 26 |

Fig. S1

1 : 10

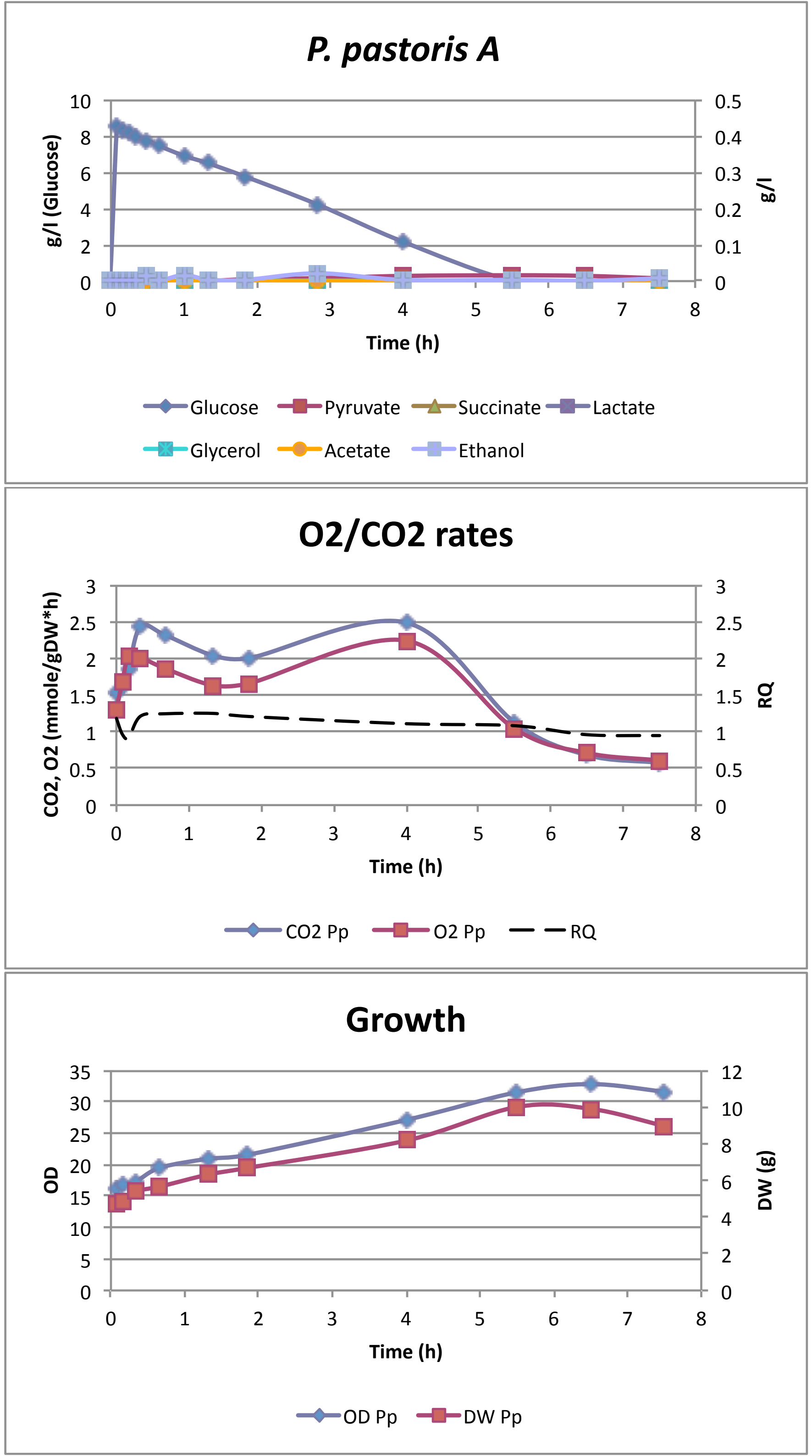
P. pastoris A
g/l (Glucose)
g/l
Time (h)
Glucose
Pyruvate
Succinate
Lactate
Glycerol
Acetate
Ethanol
O2/CO2 rates
CO2, O2 (mmole/gDW*h)
RQ
Time (h)
CO2 Pp
O2 Pp
RQ
Growth
OD
DW (g)
Time (h)
OD Pp
DW Pp

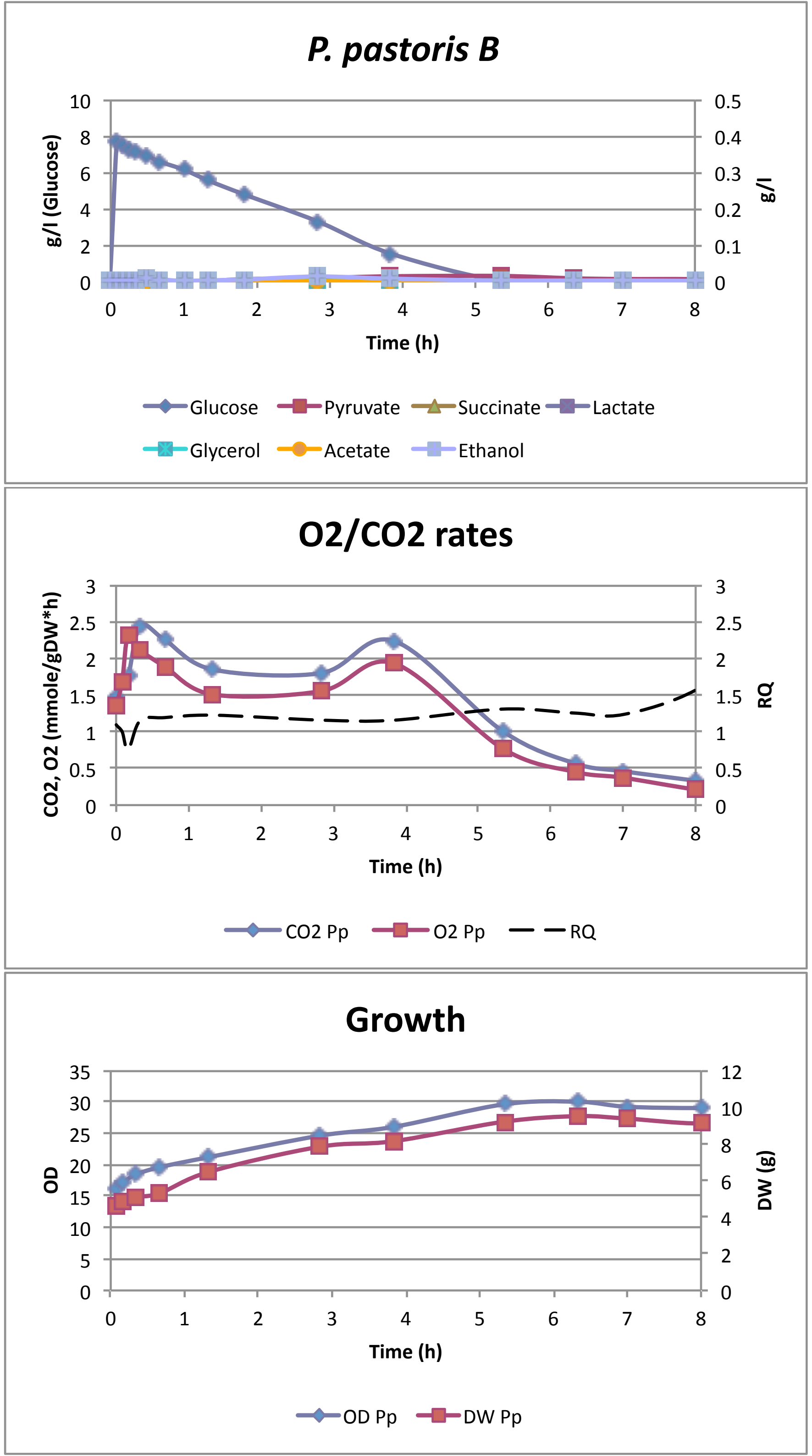
P. pastoris B
g/l (Glucose)
g/l
Time (h)
Glucose
Pyruvate
Succinate
Lactate
Glycerol
Acetate
Ethanol
O2/CO2 rates
CO2, O2 (mmole/gDW*h)
RQ
Time (h)
CO2 Pp
O2 Pp
RQ
Growth
OD
DW (g)
Time (h)
OD Pp
DW Pp

2 : 10

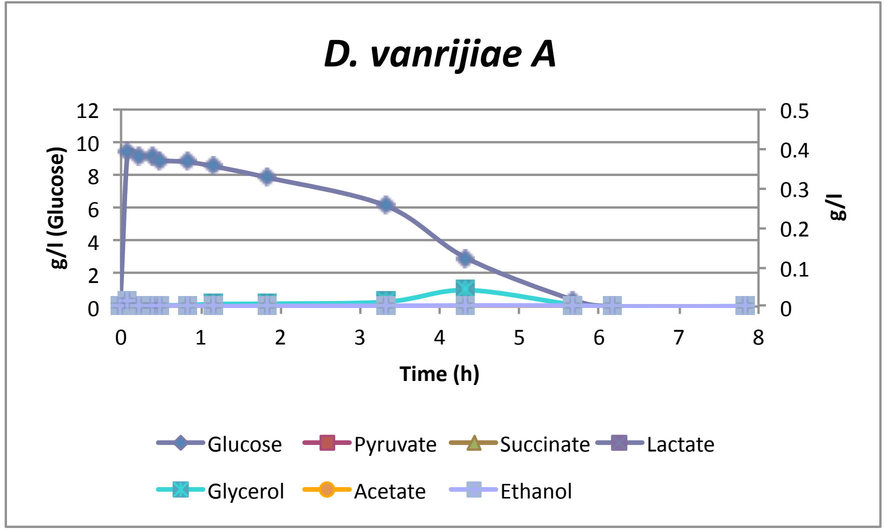

D. vanrijiae A
g/l (Glucose)
g/l
Time (h)
Glucose
Pyruvate
Succinate
Lactate
Glycerol
Acetate
Ethanol


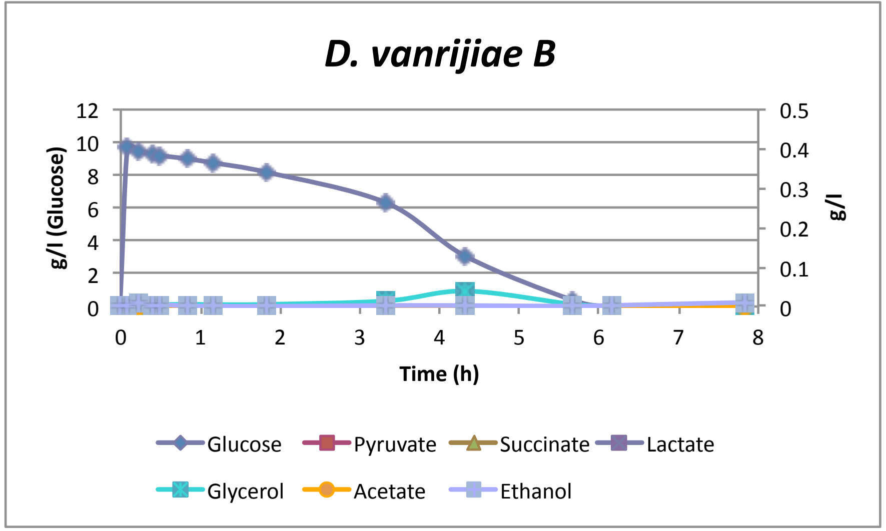

D. vanrijiae B
g/l (Glucose)
g/l
Time (h)
Glucose
Pyruvate
Succinate
Lactate
Glycerol
Acetate
Ethanol


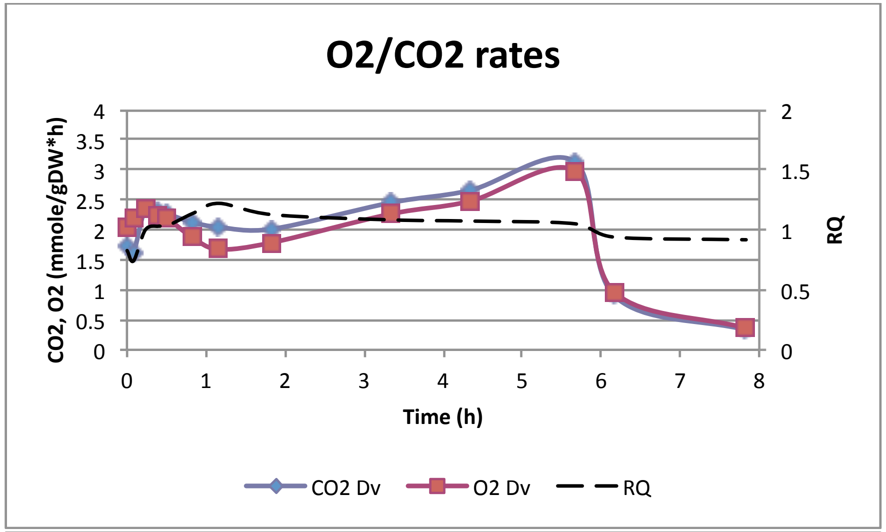

O2/CO2 rates
CO2, O2 (mmole/gDW*h)
RQ
Time (h)
CO2 Dv
O2 Dv
RQ


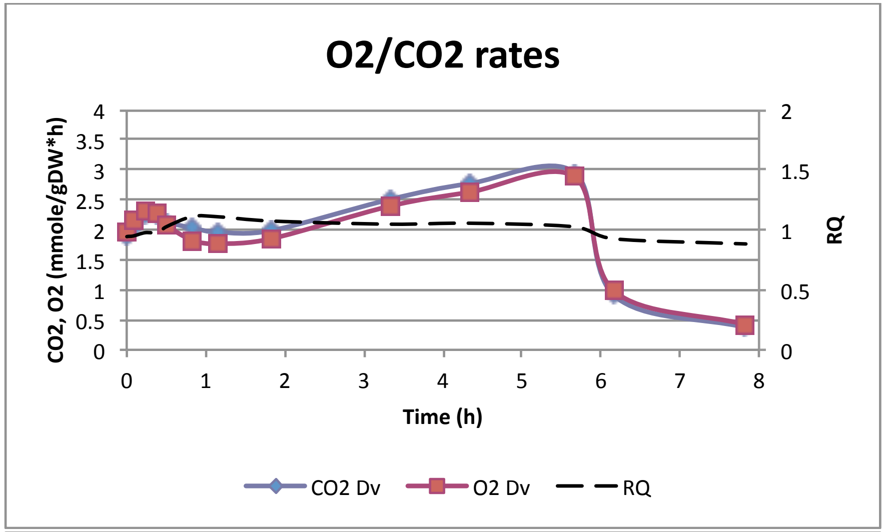

O2/CO2 rates
CO2, O2 (mmole/gDW*h)
RQ
Time (h)
CO2 Dv
O2 Dv
RQ


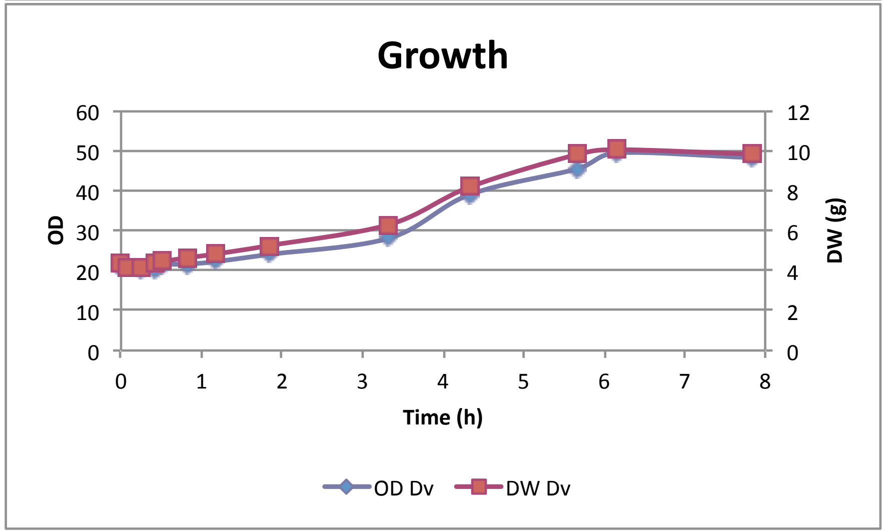

Growth
OD
DW (g)
Time (h)
OD Dv
DW Dv


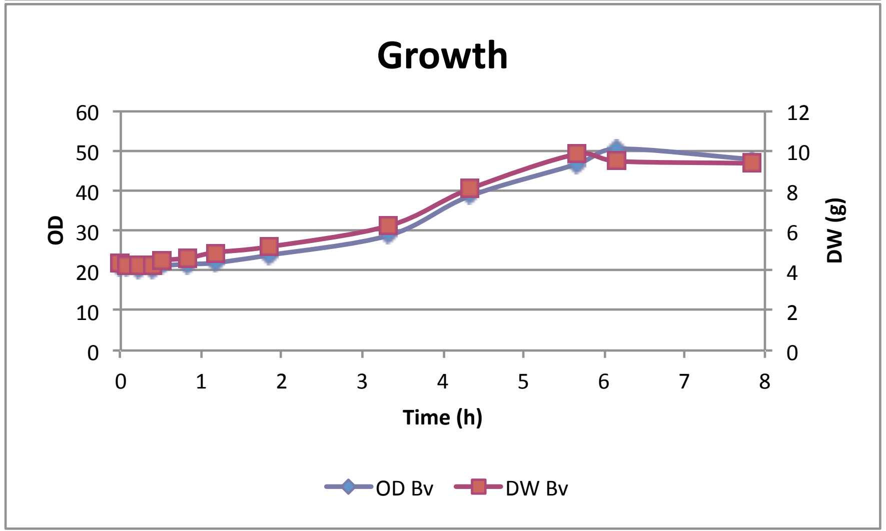

Growth
OD
DW (g)
Time (h)
OD Bv
DW Bv

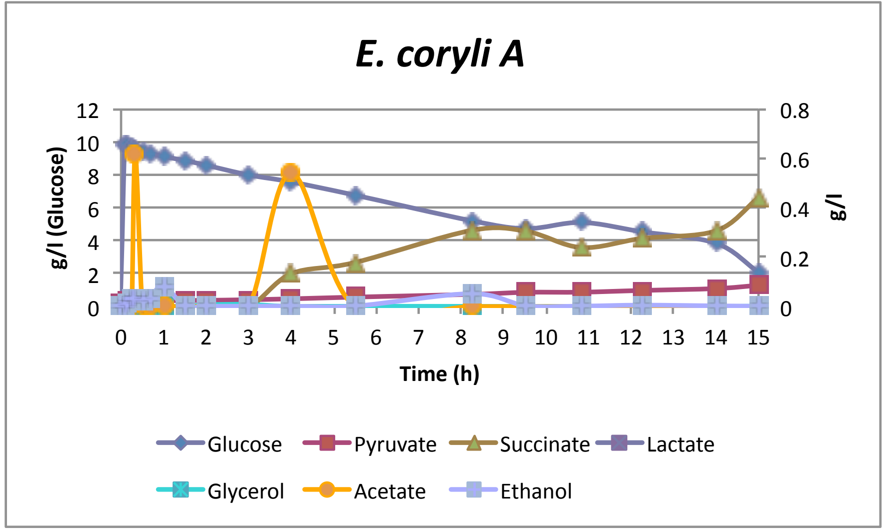
E. coryli A
g/l (Glucose)
g/l
Time (h)
Glucose
Pyruvate
Succinate
Lactate
Glycerol
Acetate
Ethanol

3 : 10

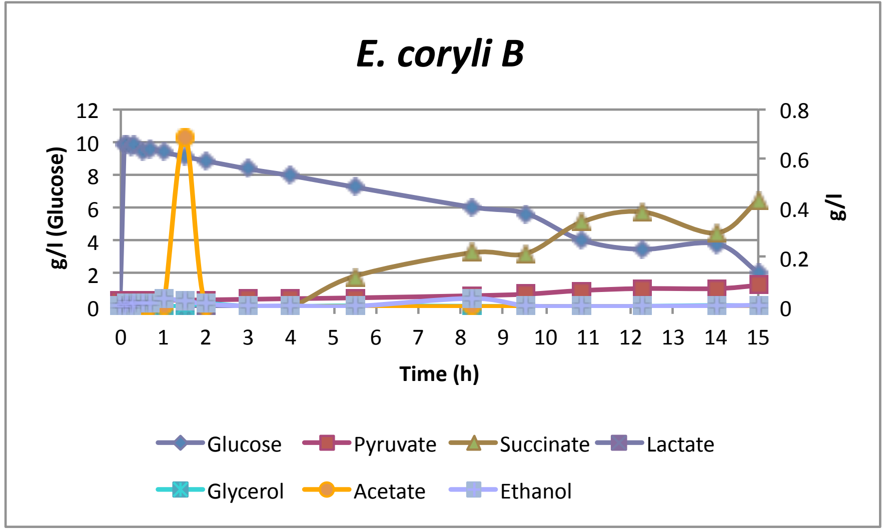
E. coryli B
g/l (Glucose)
g/l
Time (h)
Glucose
Pyruvate
Succinate
Lactate
Glycerol
Acetate
Ethanol

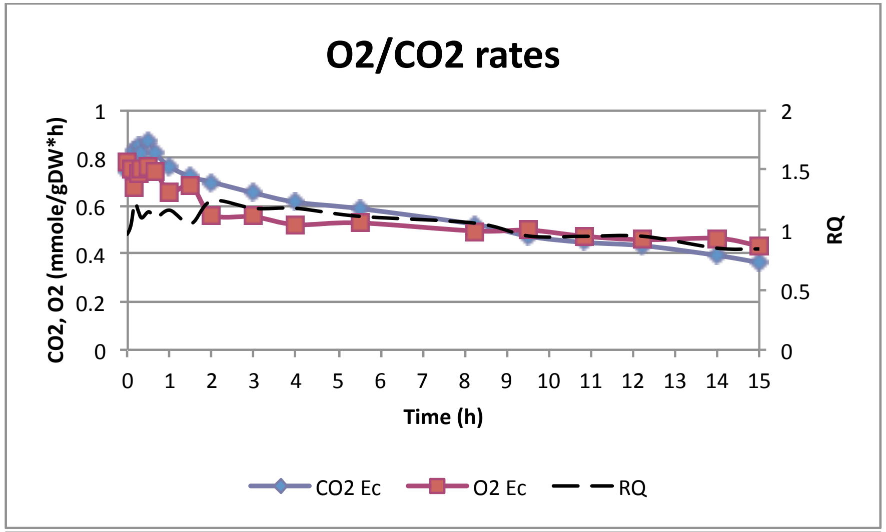
O2/CO2 rates
CO2, O2 (mmole/gDW*h)
RQ
Time (h)
CO2 Ec
O2 Ec
RQ

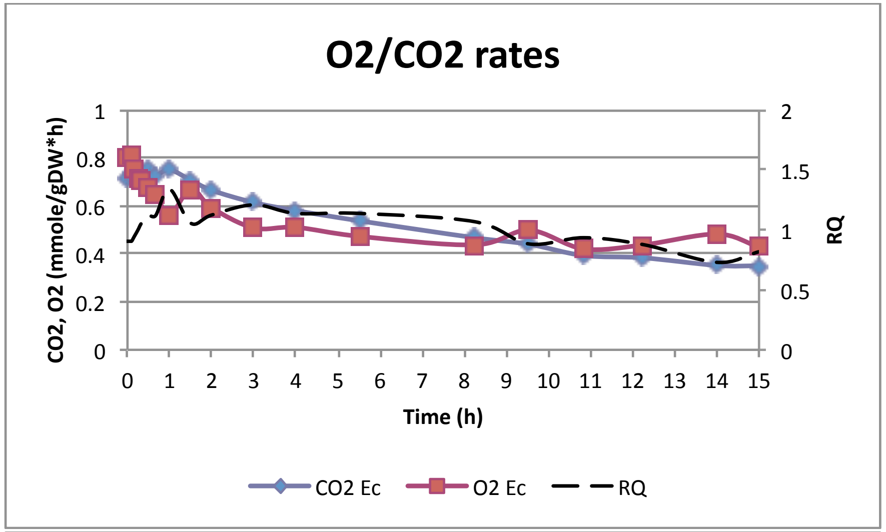
O2/CO2 rates
CO2, O2 (mmole/gDW*h)
RQ
Time (h)
CO2 Ec
O2 Ec
RQ

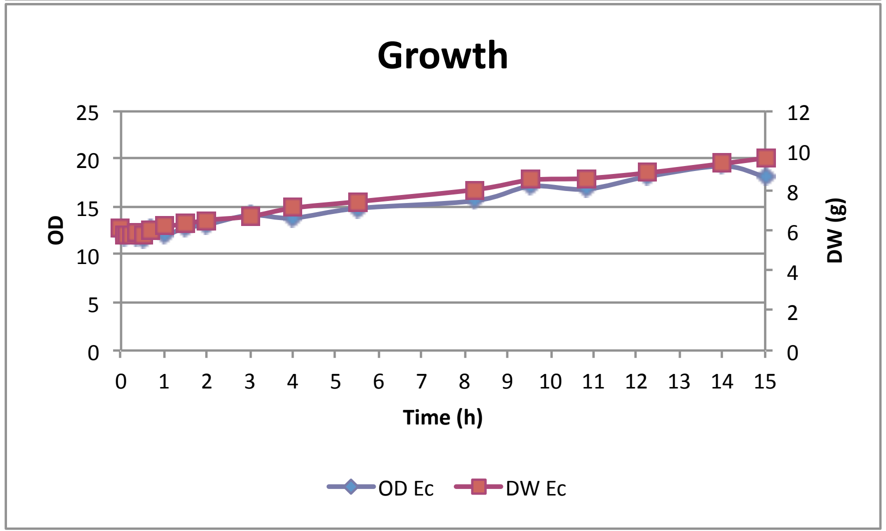
Growth
OD
DW (g)
Time (h)
OD Ec
DW Ec

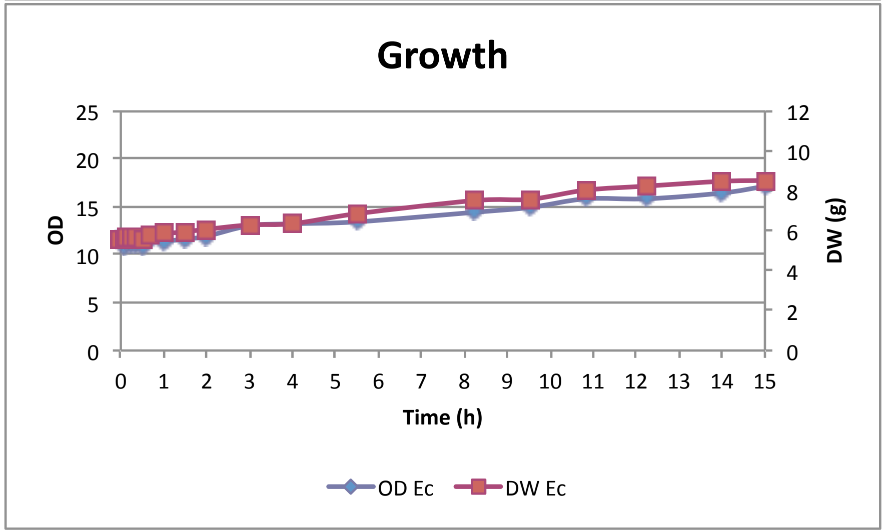
Growth
OD
DW (g)
Time (h)
OD Ec
DW Ec

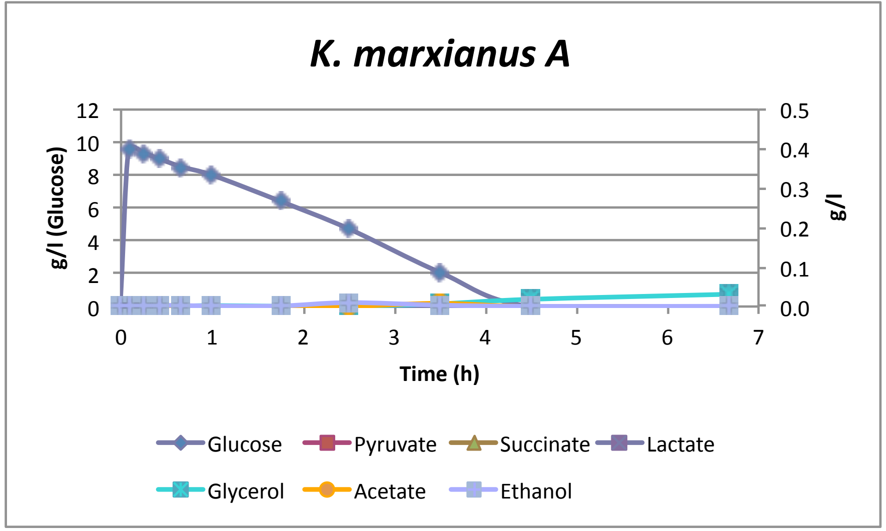
K. marxianus A
g/l (Glucose)
g/l
Time (h)
Glucose
Pyruvate
Succinate
Lactate
Glycerol
Acetate
Ethanol

4 : 10

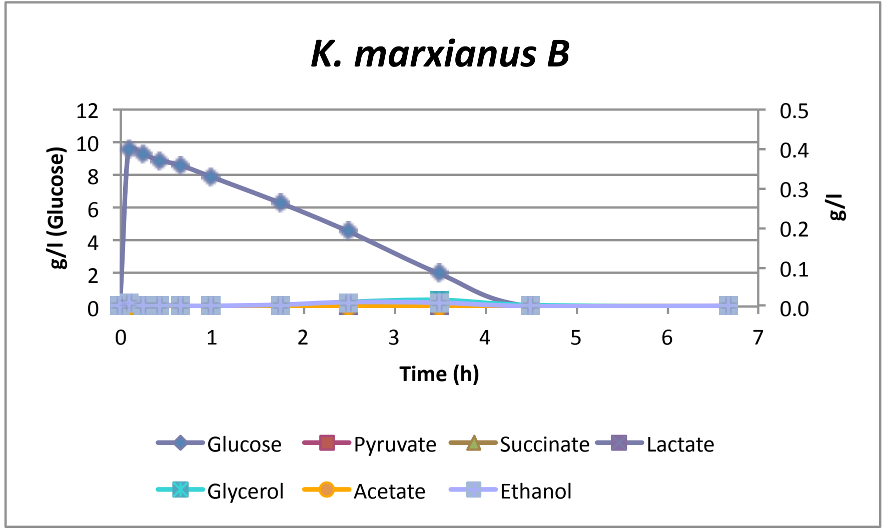
K. marxianus B
g/l (Glucose)
g/l
Time (h)
Glucose
Pyruvate
Succinate
Lactate
Glycerol
Acetate
Ethanol

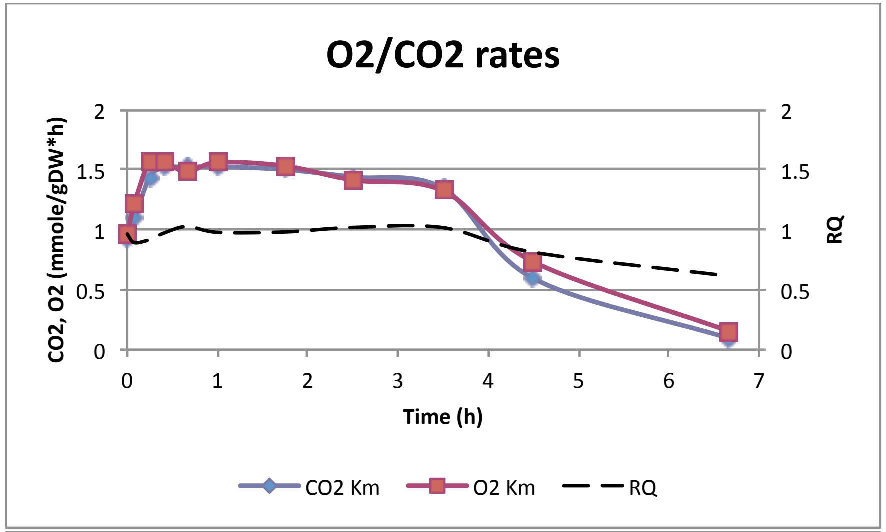
O2/CO2 rates
CO2, O2 (mmole/gDW*h)
RQ
Time (h)
CO2 Km
O2 Km
RQ

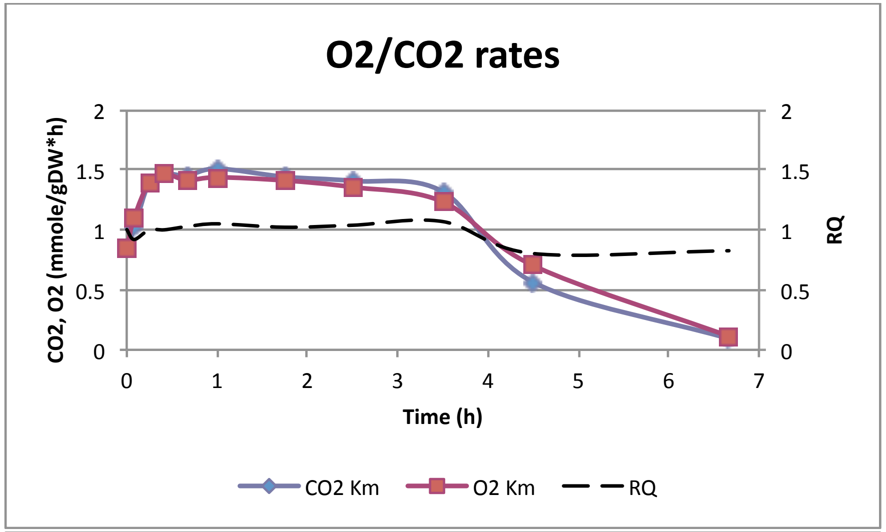
O2/CO2 rates
CO2, O2 (mmole/gDW*h)
RQ
Time (h)
CO2 Km
O2 Km
RQ

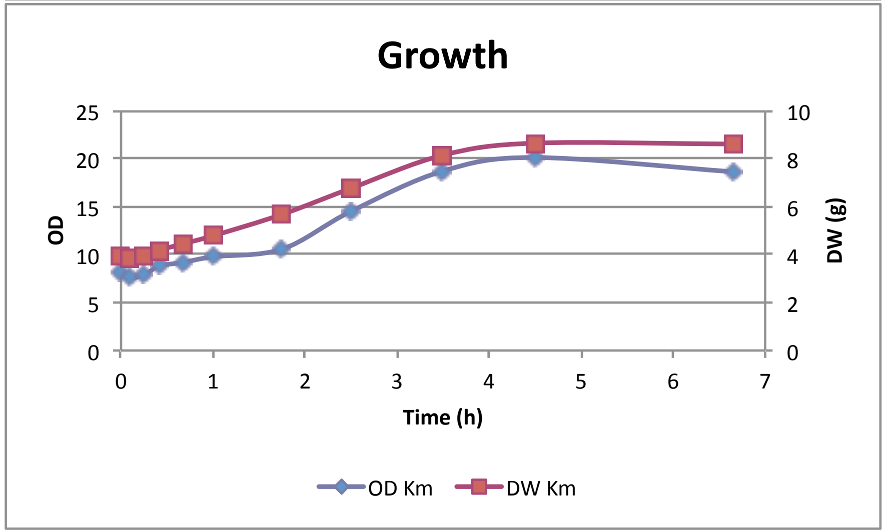
Growth
OD
DW (g)
Time (h)
OD Km
DW Km

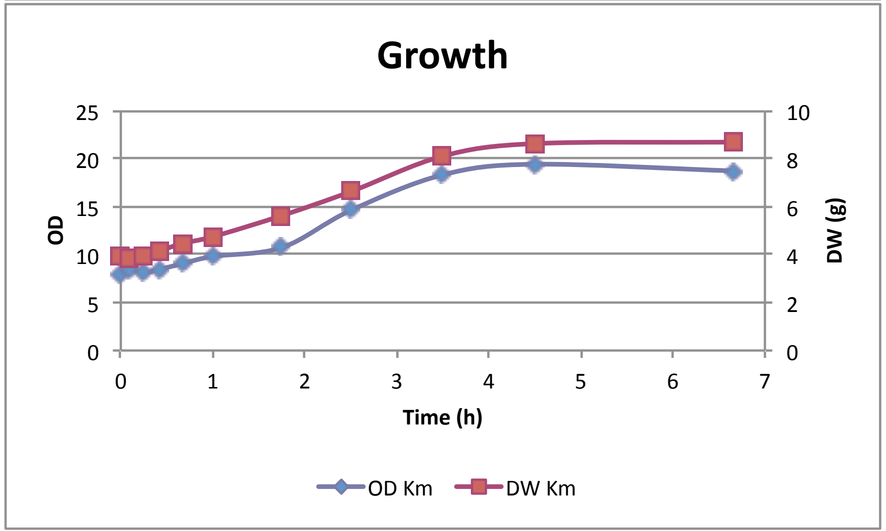
Growth
OD
DW (g)
Time (h)
OD Km
DW Km

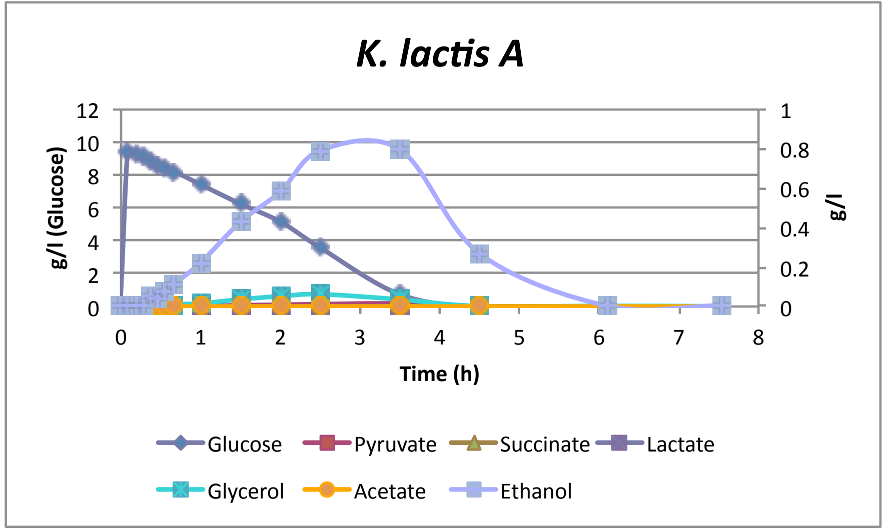
K. lactis A
g/l (Glucose)
g/l
Time (h)
Glucose
Pyruvate
Succinate
Lactate
Glycerol
Acetate
Ethanol

5 : 10

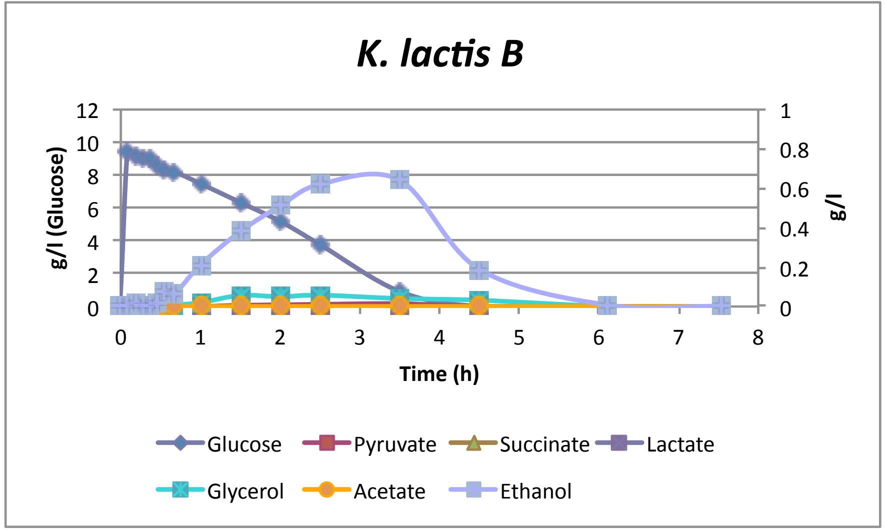
K. lactis B
g/l (Glucose)
g/l
Time (h)
Glucose
Pyruvate
Succinate
Lactate
Glycerol
Acetate
Ethanol

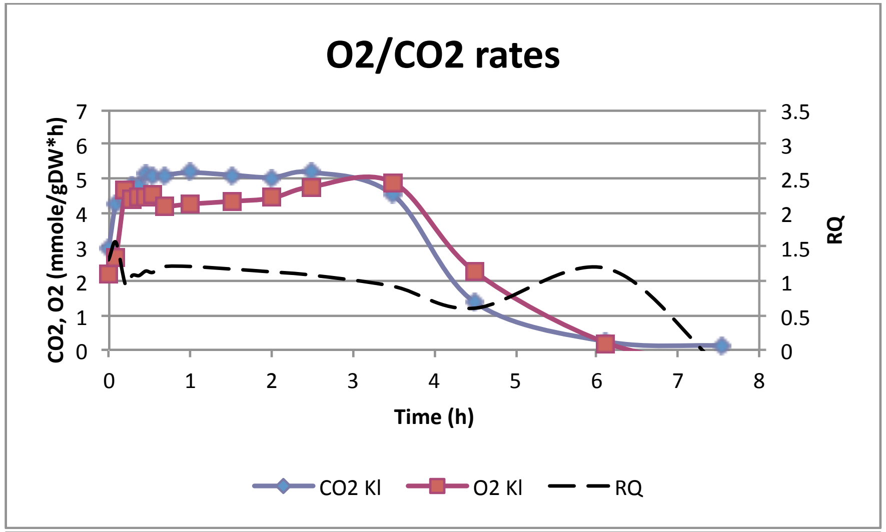
O2/CO2 rates
CO2, O2 (mmole/gDW*h)
RQ
Time (h)
CO2 Kl
O2 Kl
RQ

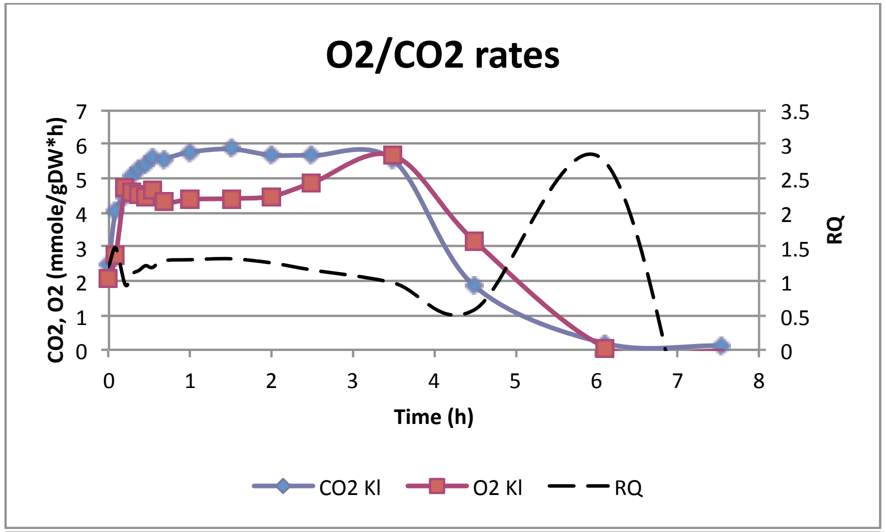
O2/CO2 rates
CO2, O2 (mmole/gDW*h)
RQ
Time (h)
CO2 Kl
O2 Kl
RQ

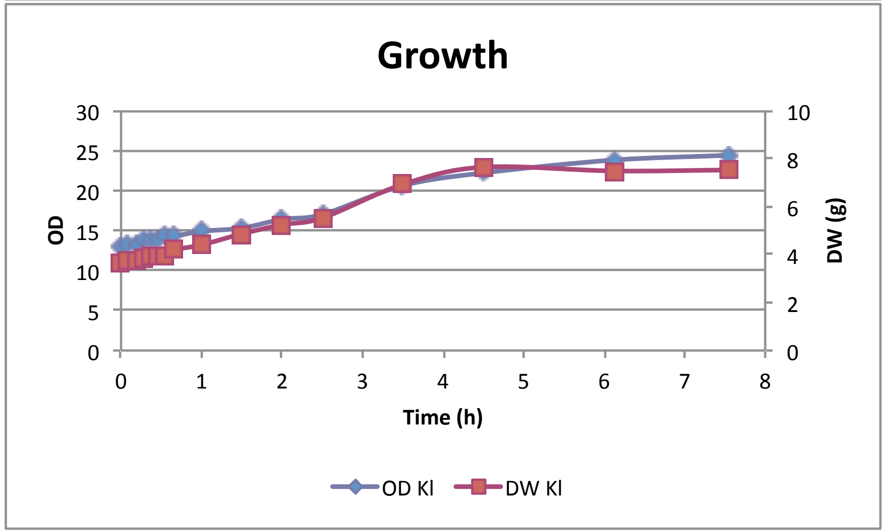
Growth
OD
DW (g)
Time (h)
OD Kl
DW Kl

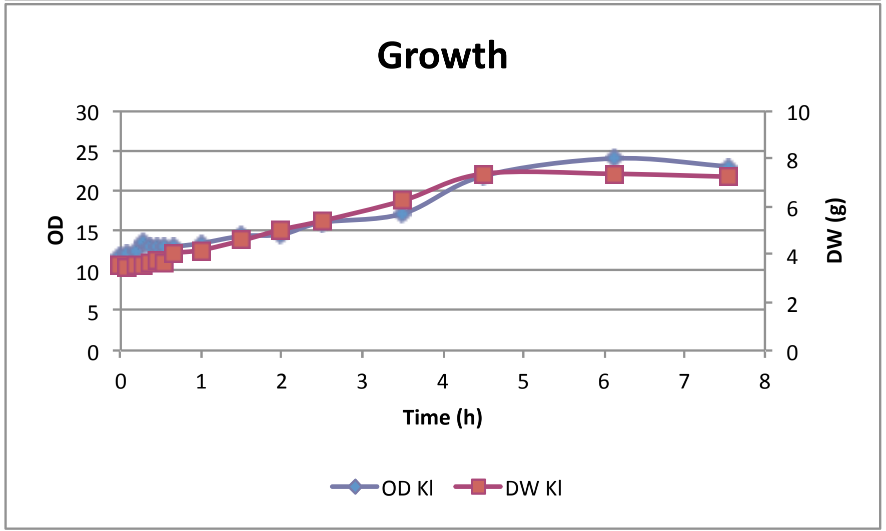
Growth
OD
DW (g)
Time (h)
OD Kl
DW Kl

6 : 10

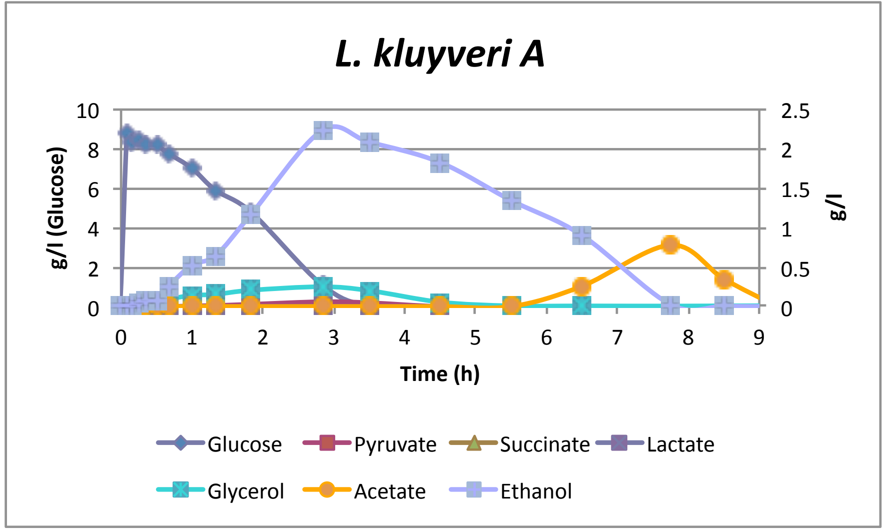
L. kluyveri A
g/l (Glucose)
g/l
Time (h)
Glucose
Pyruvate
Succinate
Lactate
Glycerol
Acetate
Ethanol

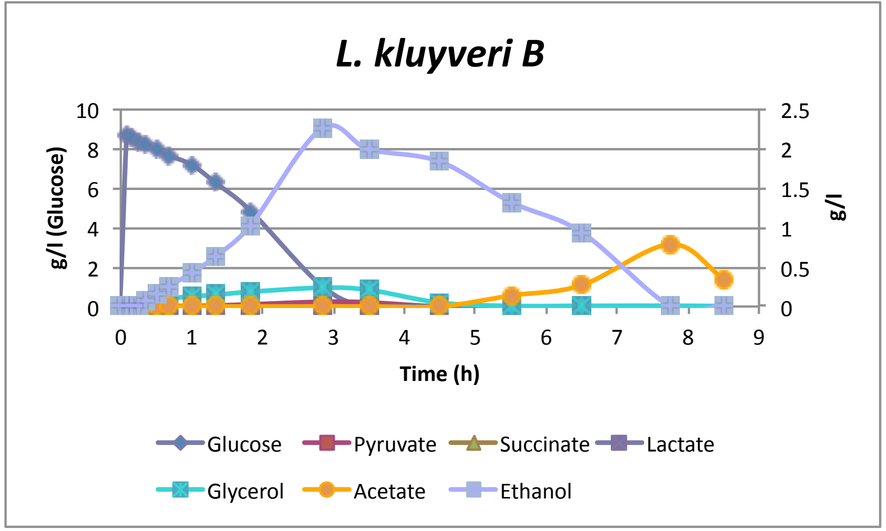
L. kluyveri B
g/l (Glucose)
g/l
Time (h)
Glucose
Pyruvate
Succinate
Lactate
Glycerol
Acetate
Ethanol

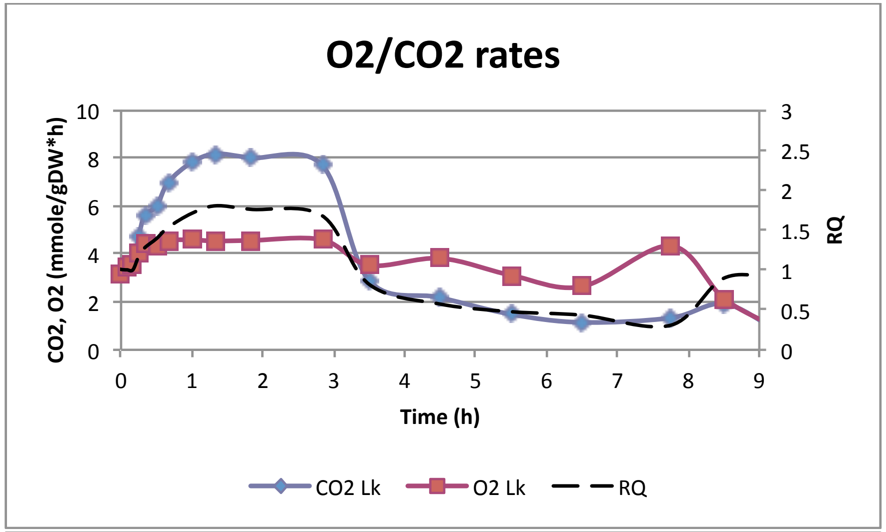
O2/CO2 rates
CO2, O2 (mmole/gDW*h)
RQ
Time (h)
CO2 Lk
O2 Lk
RQ

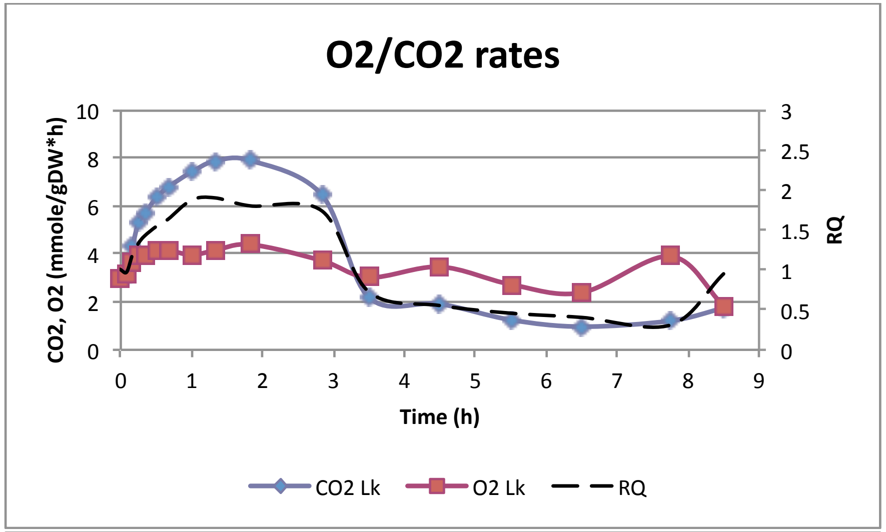
O2/CO2 rates
CO2, O2 (mmole/gDW*h)
RQ
Time (h)
CO2 Lk
O2 Lk
RQ

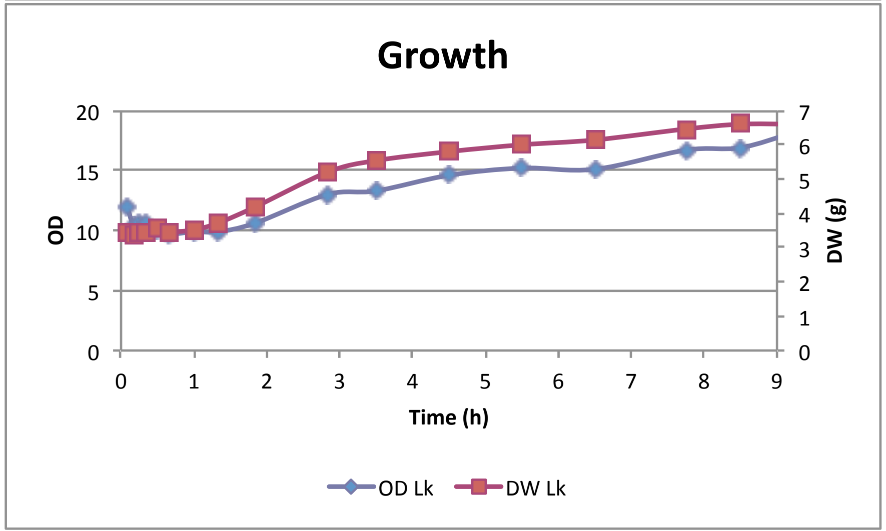
Growth
OD
DW (g)
Time (h)
OD Lk
DW Lk

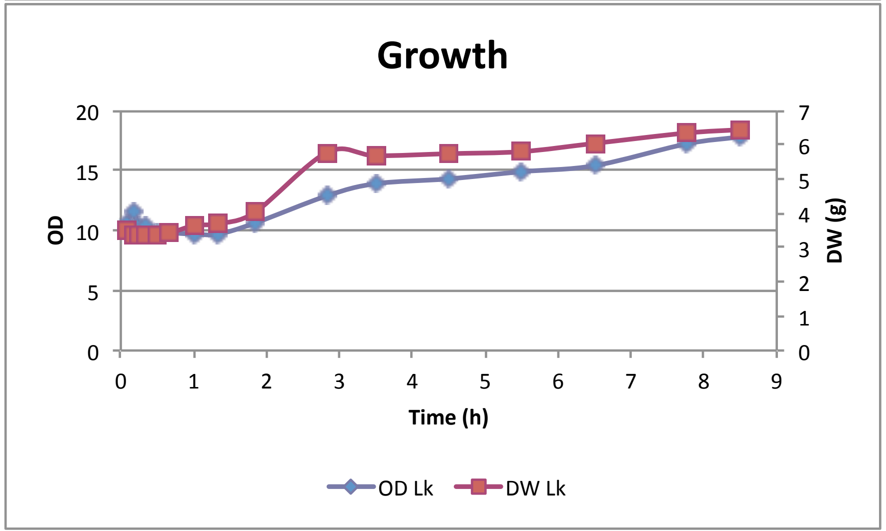
Growth
OD
DW (g)
Time (h)
OD Lk
DW Lk

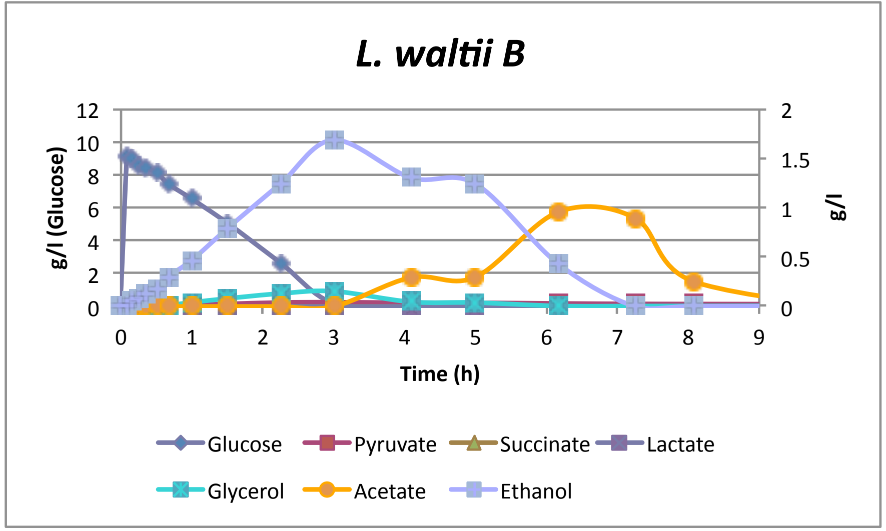
L. waltii B
g/l (Glucose)
g/l
Time (h)
Glucose
Pyruvate
Succinate
Lactate
Glycerol
Acetate
Ethanol

7 : 10

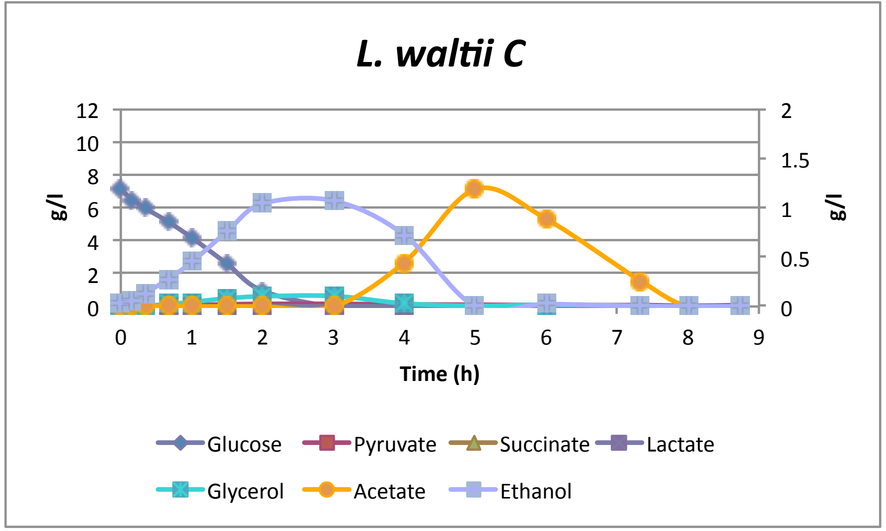
L. waltii C
g/l
g/l
Time (h)
Glucose
Pyruvate
Succinate
Lactate
Glycerol
Acetate
Ethanol

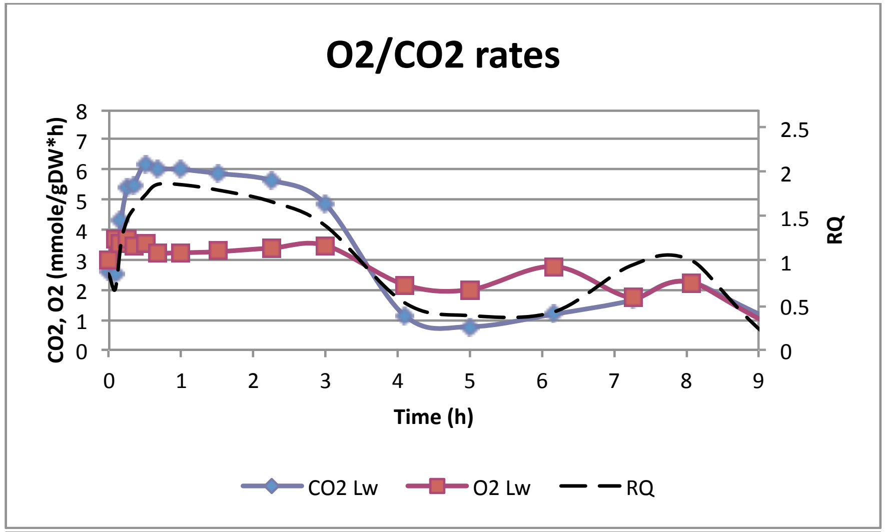
O2/CO2 rates
CO2, O2 (mmole/gDW*h)
RQ
Time (h)
CO2 Lw
O2 Lw
RQ

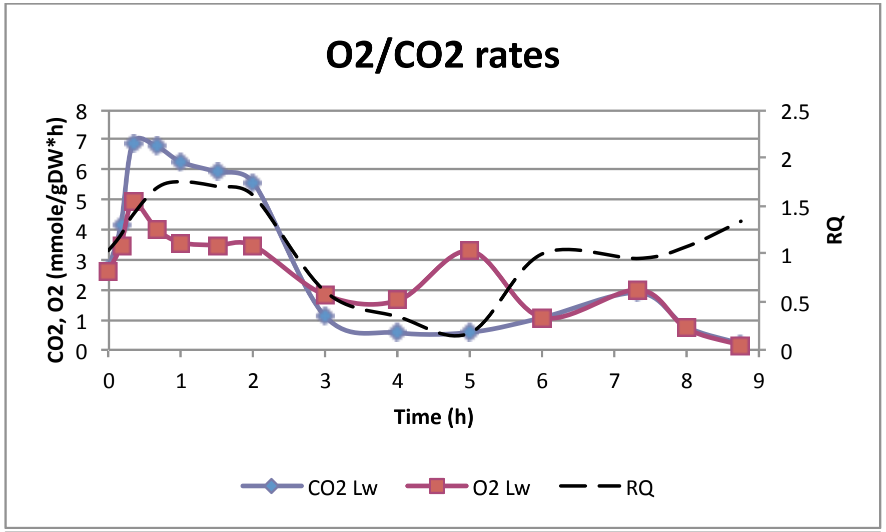
O2/CO2 rates
CO2, O2 (mmole/gDW*h)
RQ
Time (h)
CO2 Lw
O2 Lw
RQ

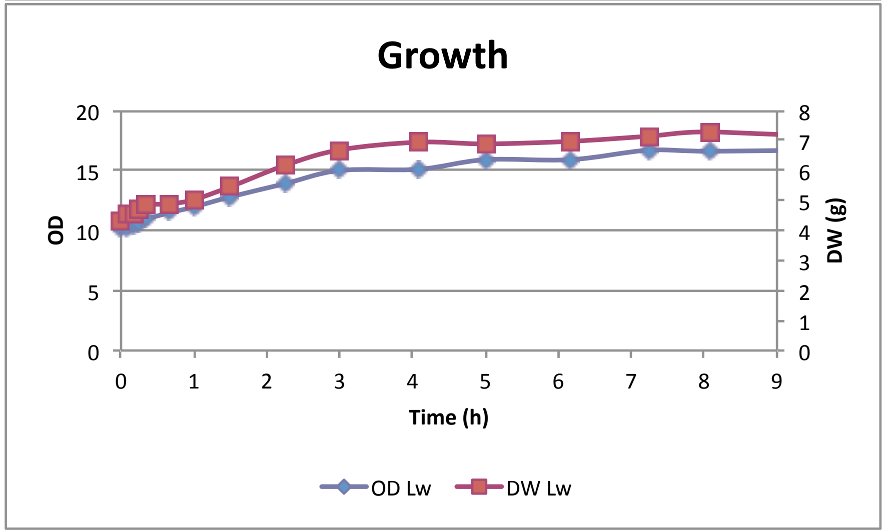
Growth
OD
DW (g)
Time (h)
OD Lw
DW Lw

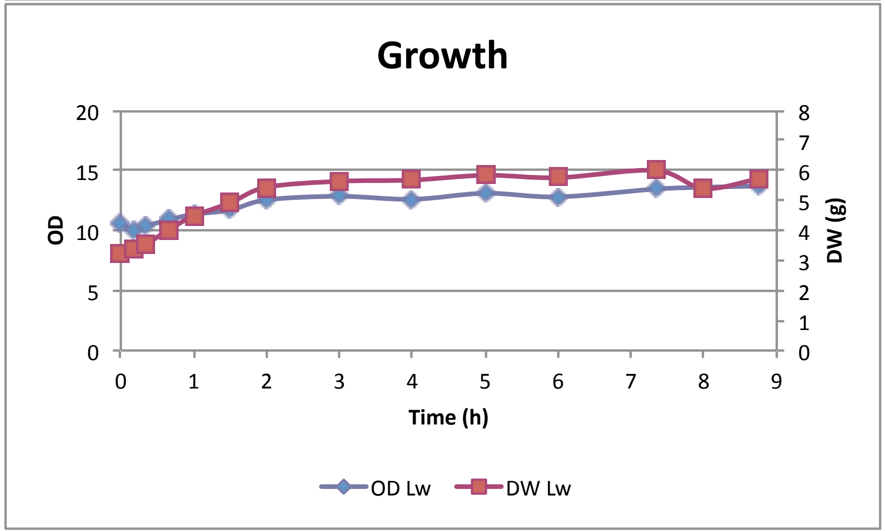
Growth
OD
DW (g)
Time (h)
OD Lw
DW Lw

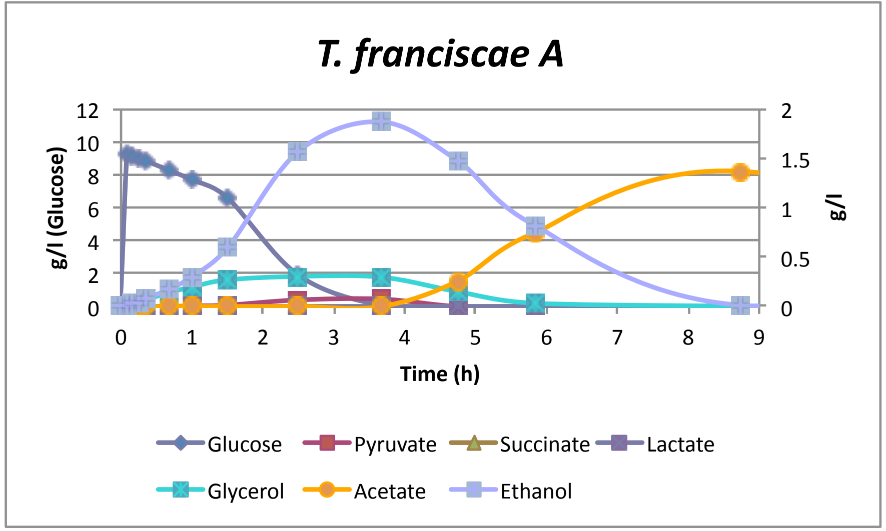
T. franciscae A
g/l (Glucose)
g/l
Time (h)
Glucose
Pyruvate
Succinate
Lactate
Glycerol
Acetate
Ethanol

8 : 10

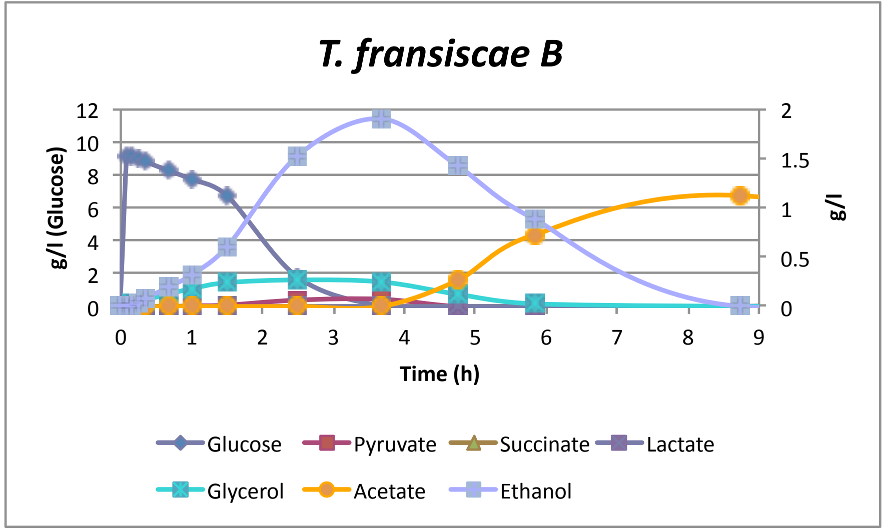
T. fransiscae B
g/l (Glucose)
g/l
Time (h)
Glucose
Pyruvate
Succinate
Lactate
Glycerol
Acetate
Ethanol

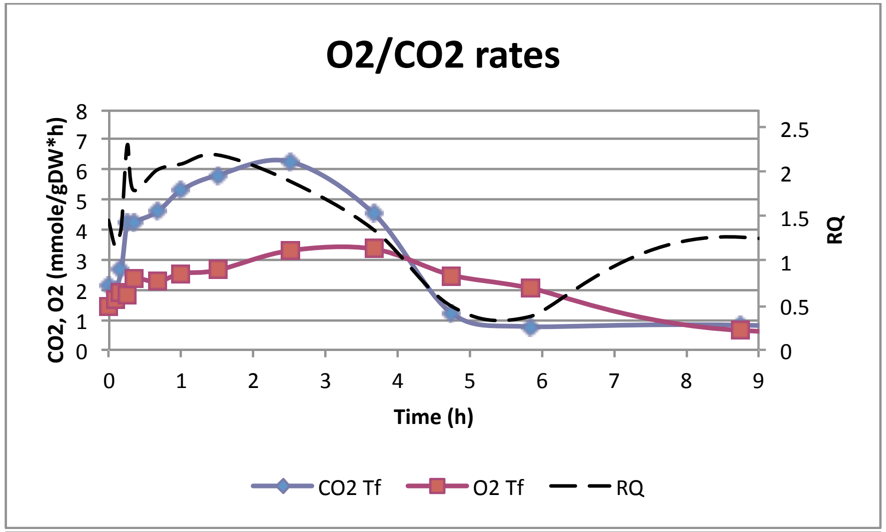
O2/CO2 rates
CO2, O2 (mmole/gDW*h)
RQ
Time (h)
CO2 Tf
O2 Tf
RQ

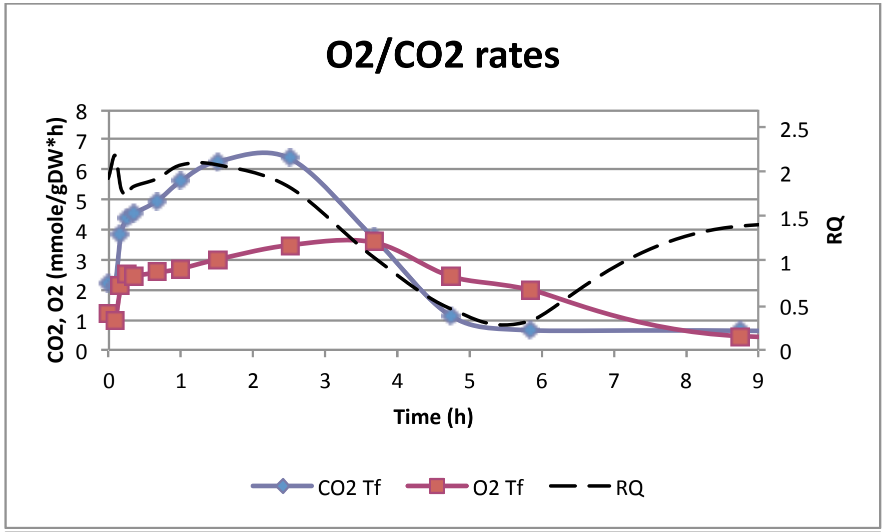
O2/CO2 rates
CO2, O2 (mmole/gDW*h)
RQ
Time (h)
CO2 Tf
O2 Tf
RQ

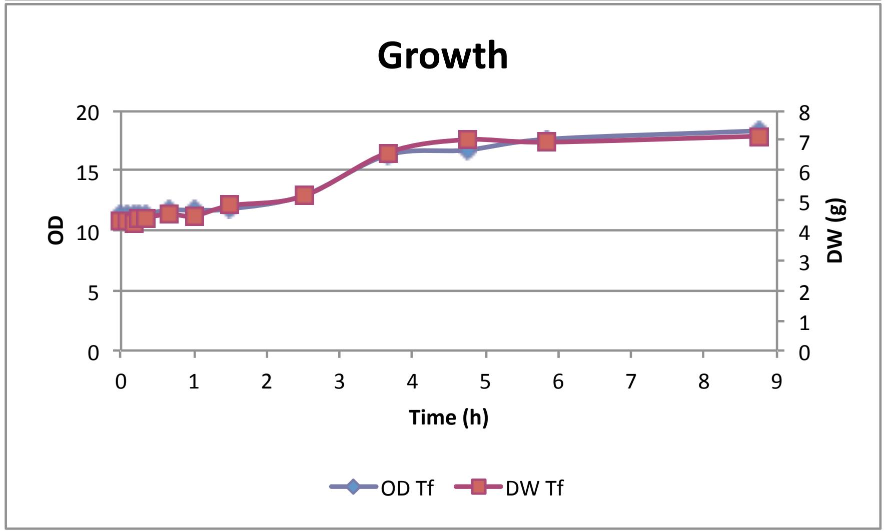
Growth
OD
DW (g)
Time (h)
OD Tf
DW Tf

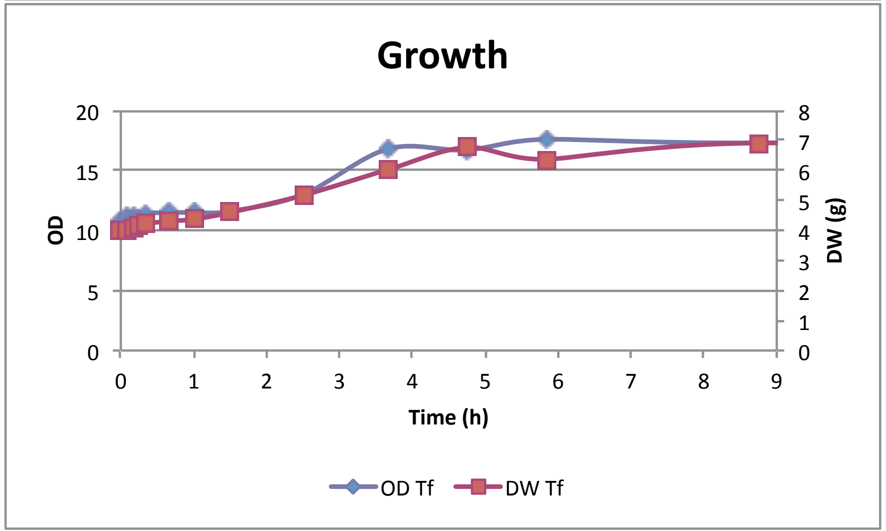
Growth
OD
DW (g)
Time (h)
OD Tf
DW Tf

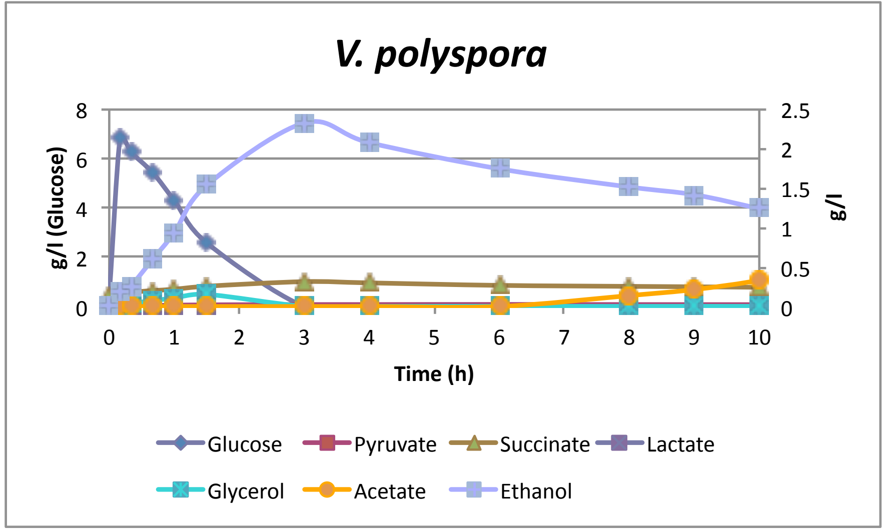
V. polyspora
g/l (Glucose)
g/l
Time (h)
Glucose
Pyruvate
Succinate
Lactate
Glycerol
Acetate
Ethanol

9 : 10

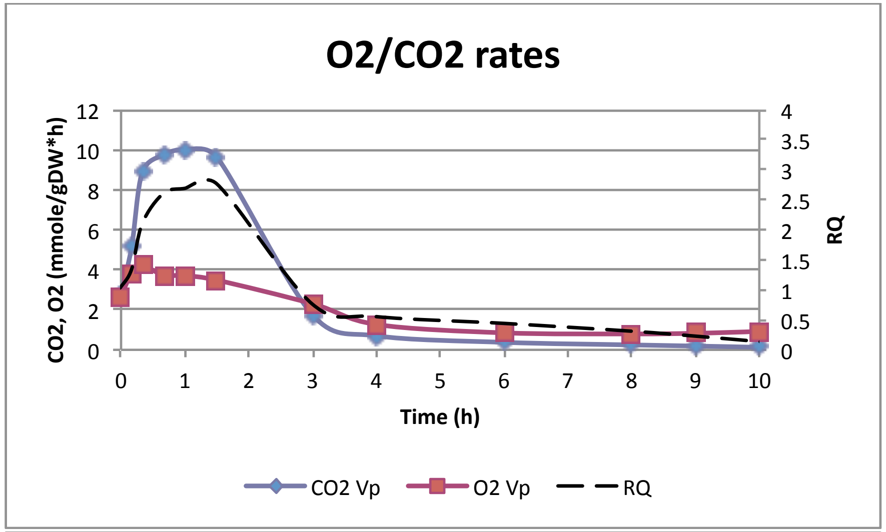
O2/CO2 rates
CO2, O2 (mmole/gDW*h)
RQ
Time (h)
CO2 Vp
O2 Vp
RQ

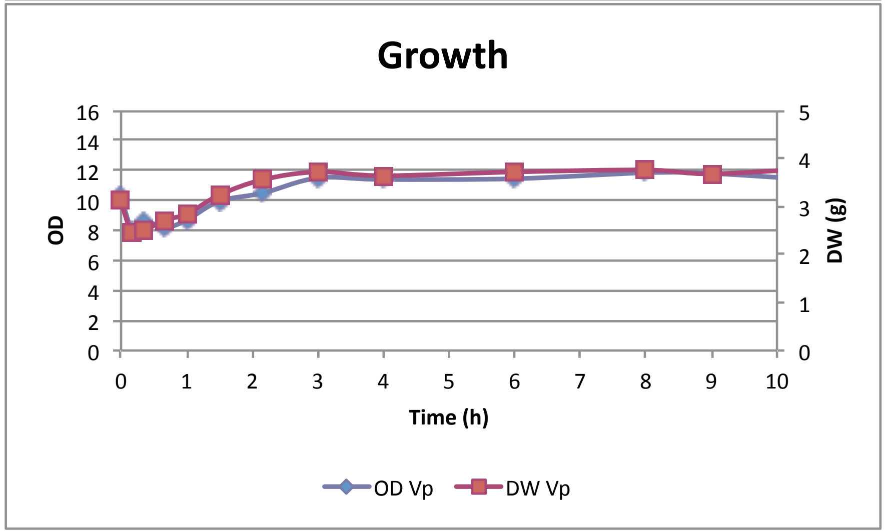
Growth
OD
DW (g)
Time (h)
OD Vp
DW Vp

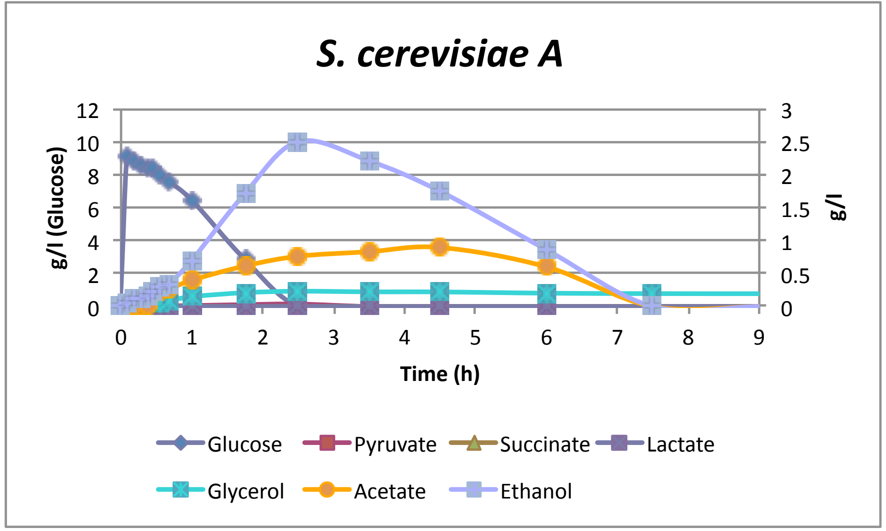
S. cerevisiae A
g/l (Glucose)
g/l
Time (h)
Glucose
Pyruvate
Succinate
Lactate
Glycerol
Acetate
Ethanol

10 : 10

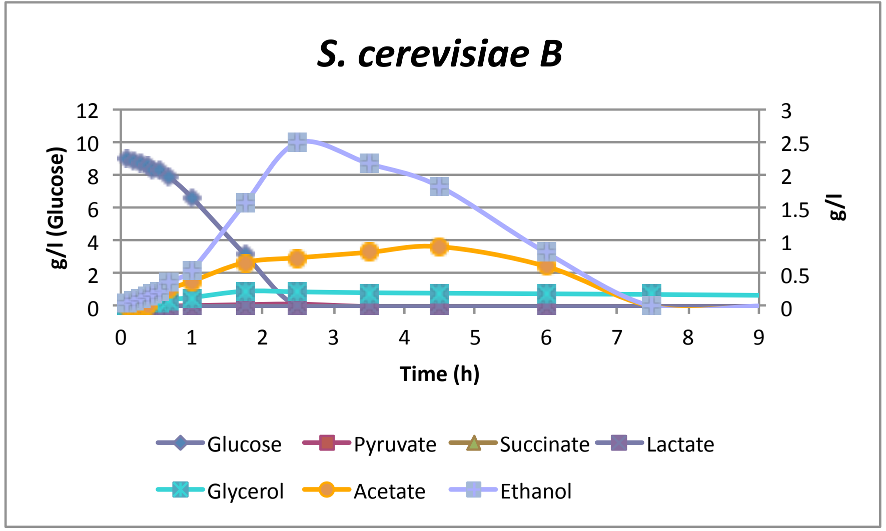
S. cerevisiae B
g/l (Glucose)
g/l
Time (h)
Glucose
Pyruvate
Succinate
Lactate
Glycerol
Acetate
Ethanol

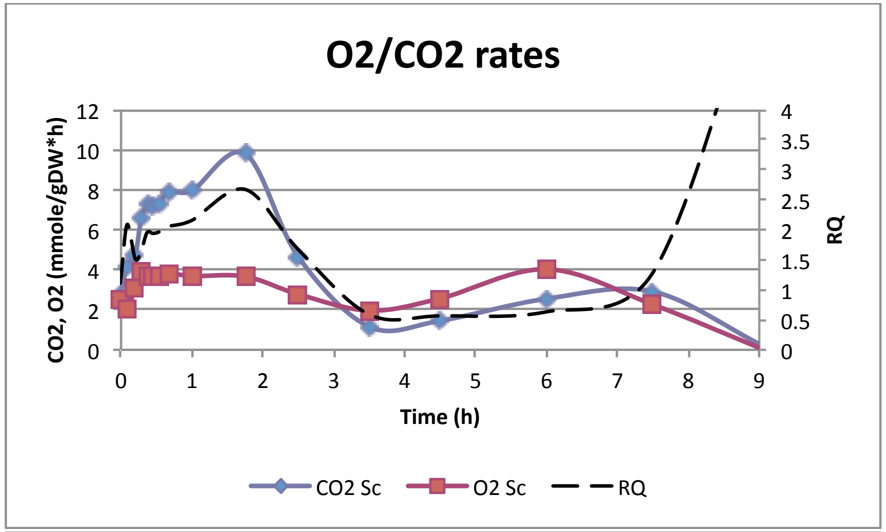
O2/CO2 rates
CO2, O2 (mmole/gDW*h)
RQ
Time (h)
CO2 Sc
O2 Sc
RQ

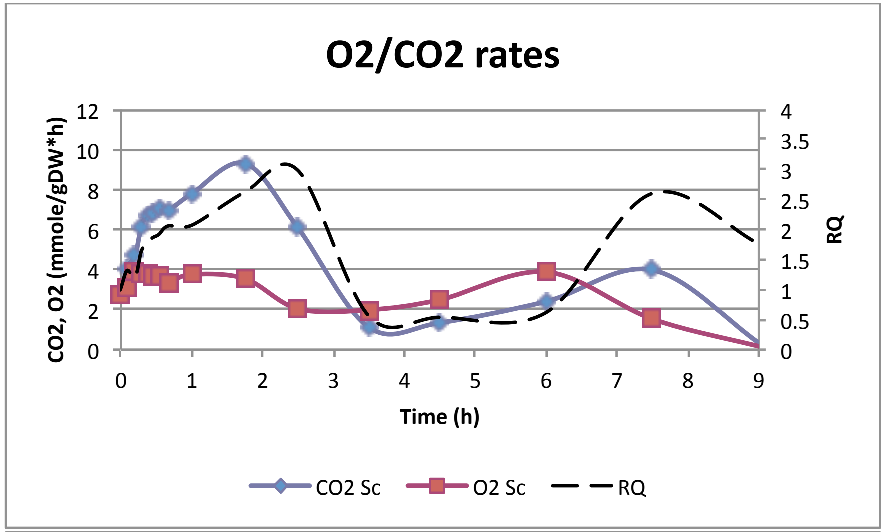
O2/CO2 rates
CO2, O2 (mmole/gDW*h)
RQ
Time (h)
CO2 Sc
O2 Sc
RQ

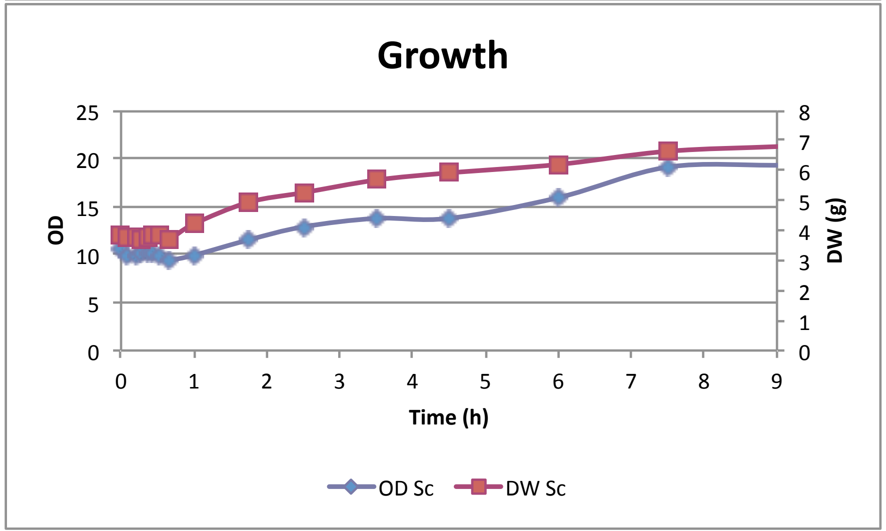
Growth
OD
DW (g)
Time (h)
OD Sc
DW Sc

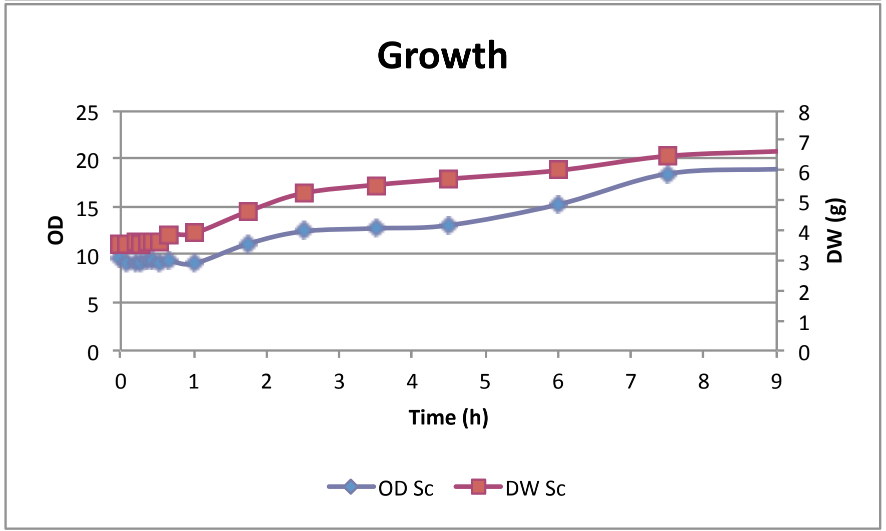
Growth
OD
DW (g)
Time (h)
OD Sc
DW Sc

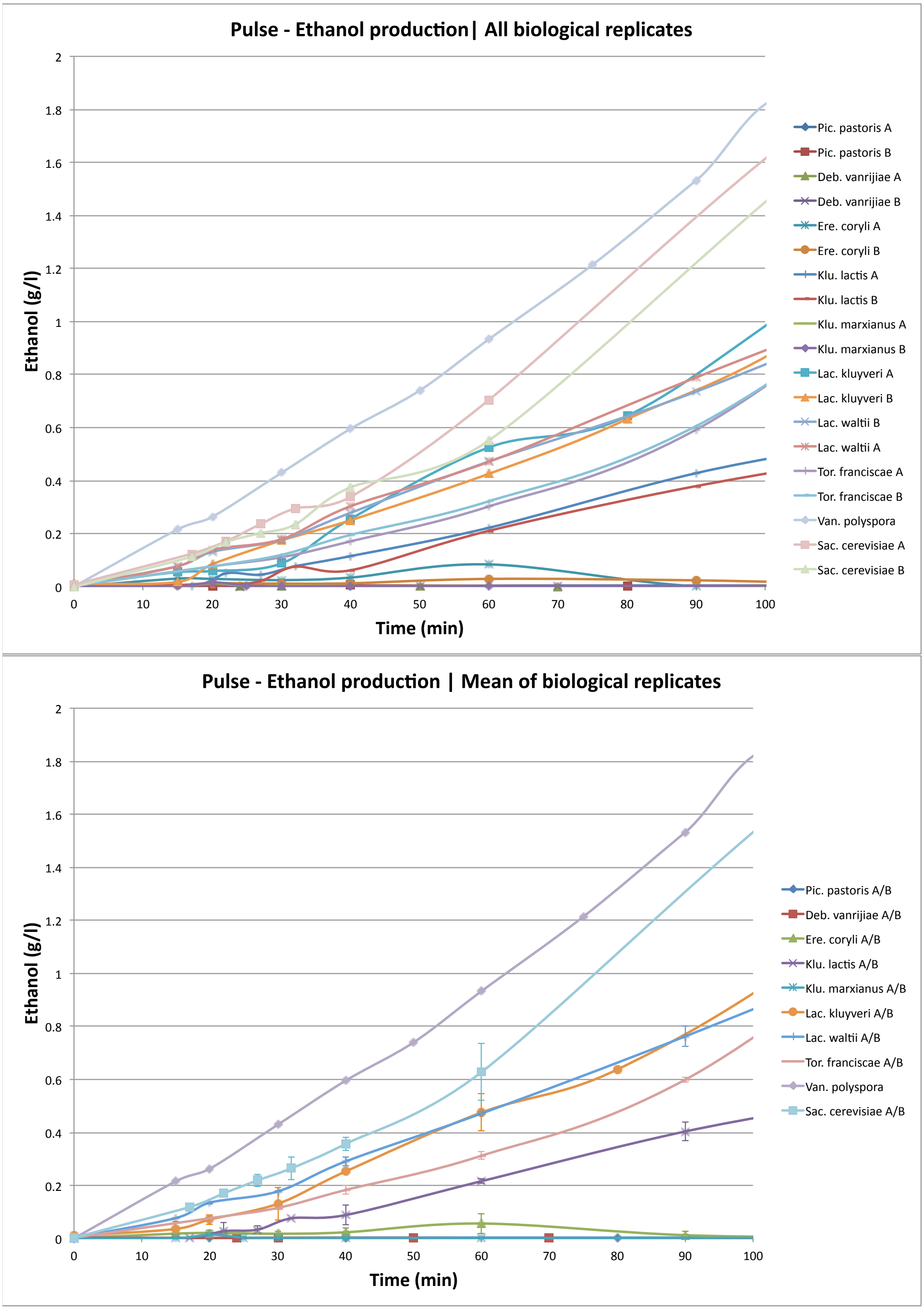


**Figure S2: Ethanol formation in response to a glucose pulse.** Short-term Crabtree effect is defined as instantaneous (within minutes) ethanol formation of aerobic steady-state cultures, when being shifted from growth in glucose limited to glucose excess conditions. 10 yeast species that belong to the order *Saccharomycetales* were investigated in duplicate for the short-term Crabtree effect. Our results reveal that six yeast species, *S. cerevisiae, V. polyspora, T. fransiscae, L. waltii and L. kluyveri* exhibit strong short-term Crabtree effect, exhibited

significant ethanol formation within 10 minutes. *K. lactis* has previously been characterized as an intermediate with an invariable ethanol formation within 40-50 minutes [1], but we could detect significant amount of ethanol within 20 minutes in our experiments. The response to glucose appears slow in *K. lactis,* which is most likely due to low initial ethanol production rates that result in ethanol concentrations below detection limit (see also figure 1). The remaining yeast species, *K. marxianus, E. coryli, D. vanrijiae* and *P. pastoris* do not form any significant amount of ethanol in response to a glucose-pulse. The average ethanol formation of two biological replicates, with error bars corresponding to the standard deviation, is shown.

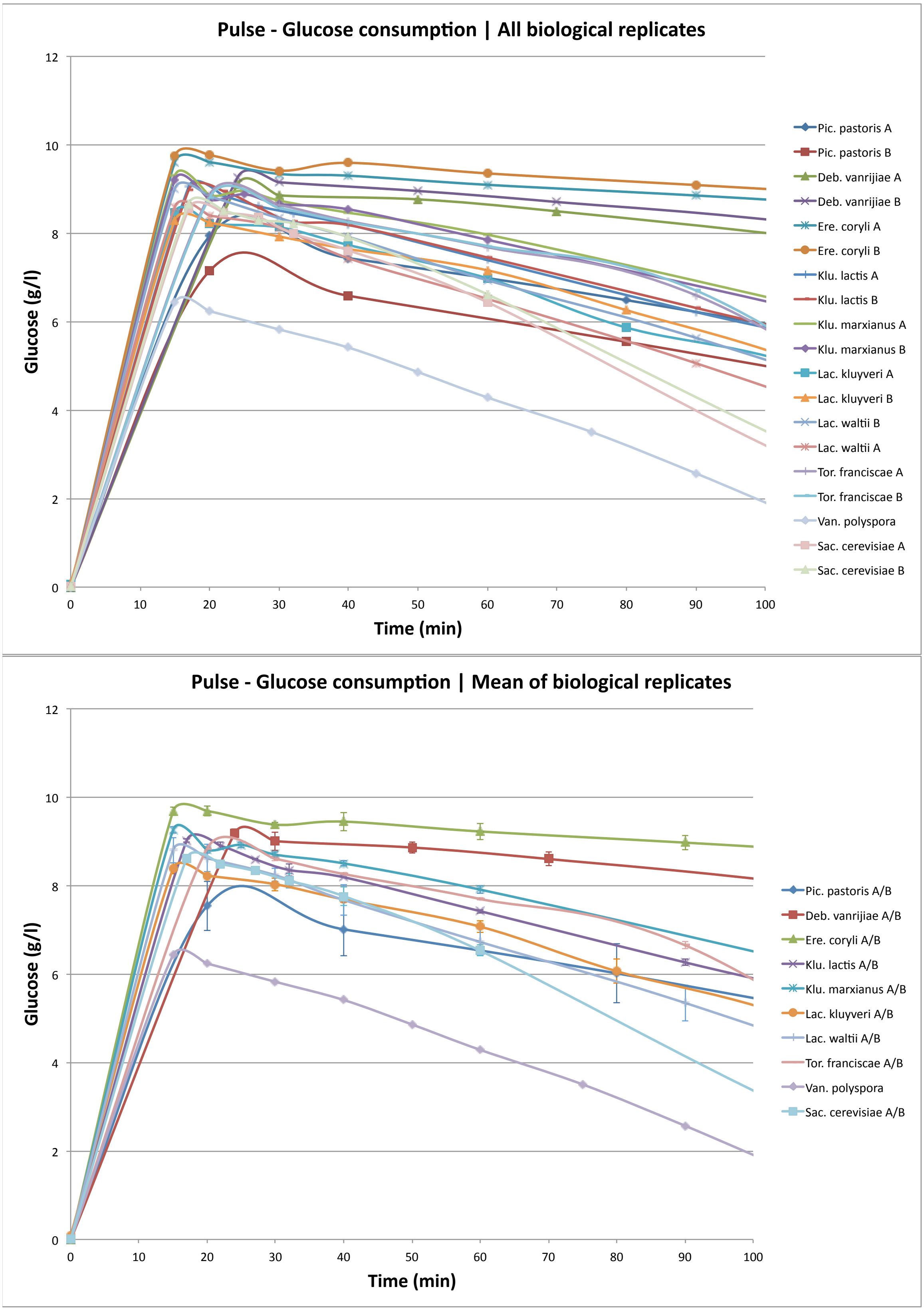


**Figure S3: Continuous glucose consumption in response to a glucose-pulse.** All yeast species continued to consume glucose after a glucose-pulse. The average glucose consumption from two biological replicates with error bars corresponding to one standard deviation are shown.

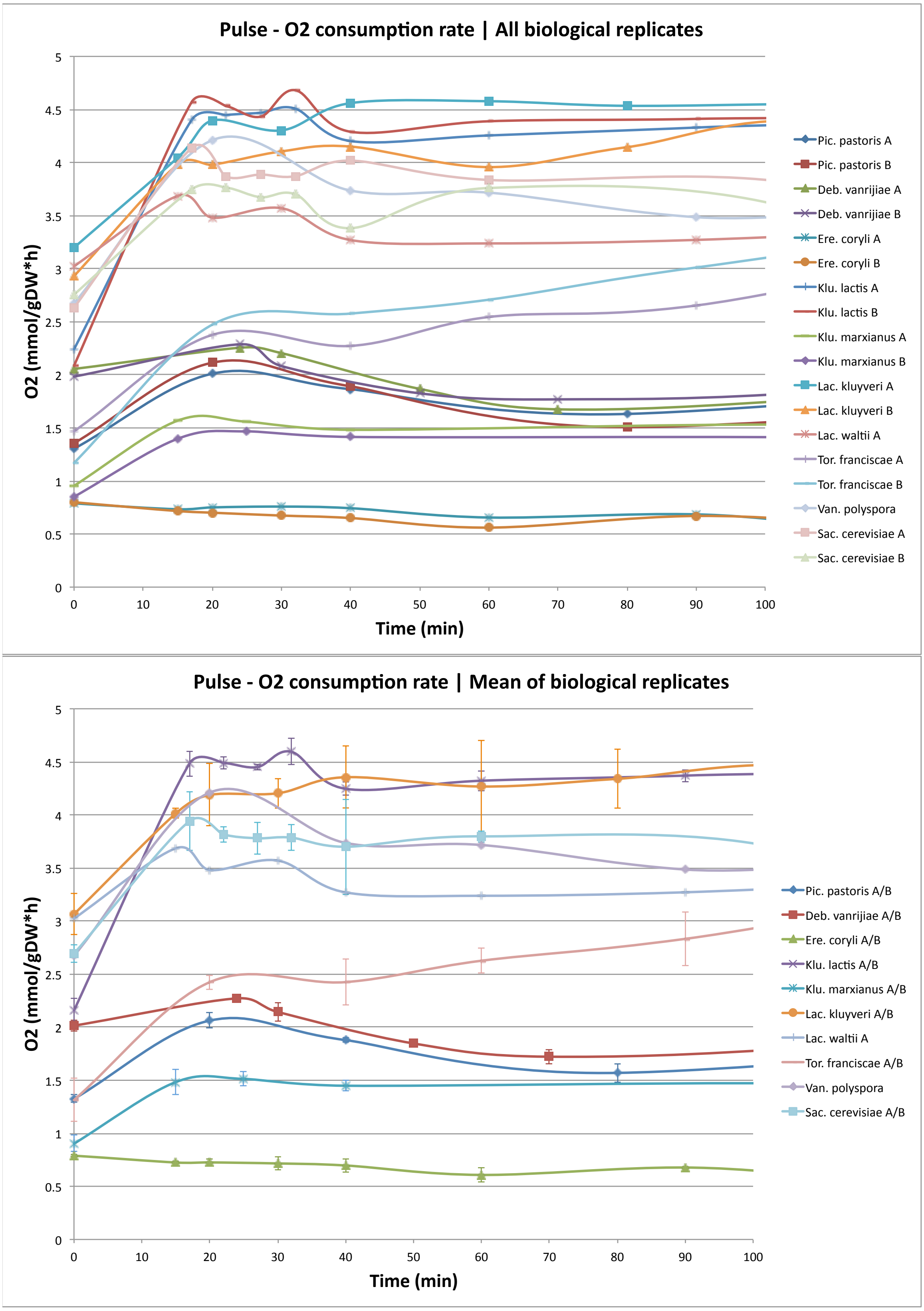


**Figure S4: Oxygen consumption rates (first 100 min).** Yeast species were studied for their oxygen consumption rates, and all the time points during the first 100 minutes after a glucose-pulse are illustrated. The average oxygen consumption rates of two biological replicates for each time point, with error bars corresponding to the standard deviation, are shown.

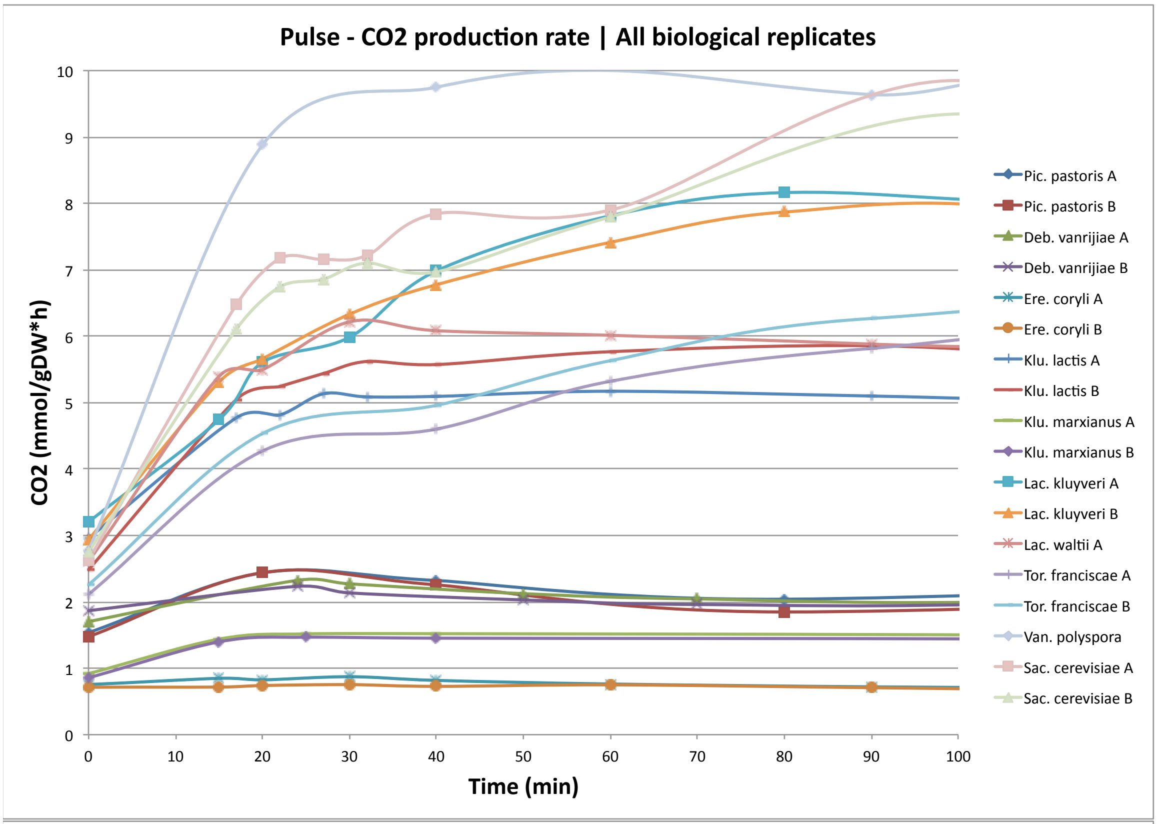


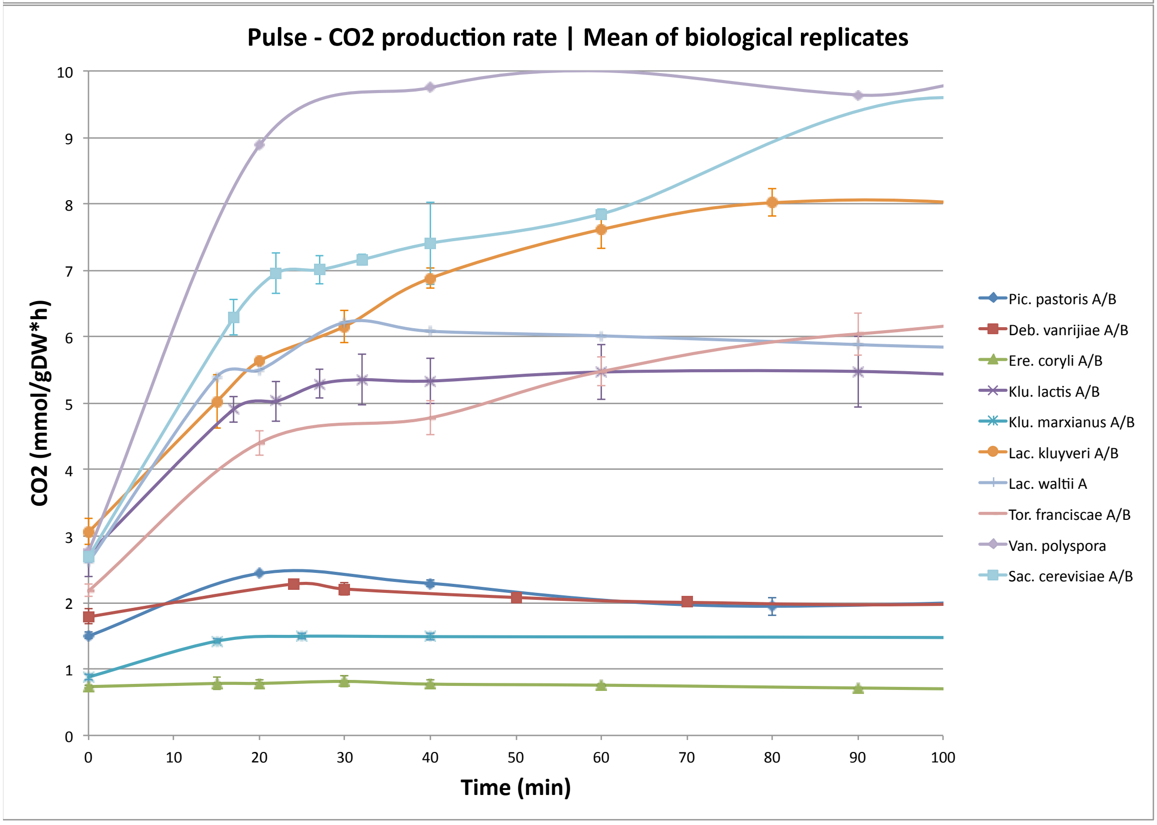


**Figure S5: Carbon dioxide production rates (first 100 min).** Yeast species were studied for their carbon dioxide production rates, and all the time points during the first 100 minutes after a glucose pulse are illustrated. The average carbon dioxide production rates of two biological replicates for each time point, with error bars corresponding to the standard deviation, are shown.

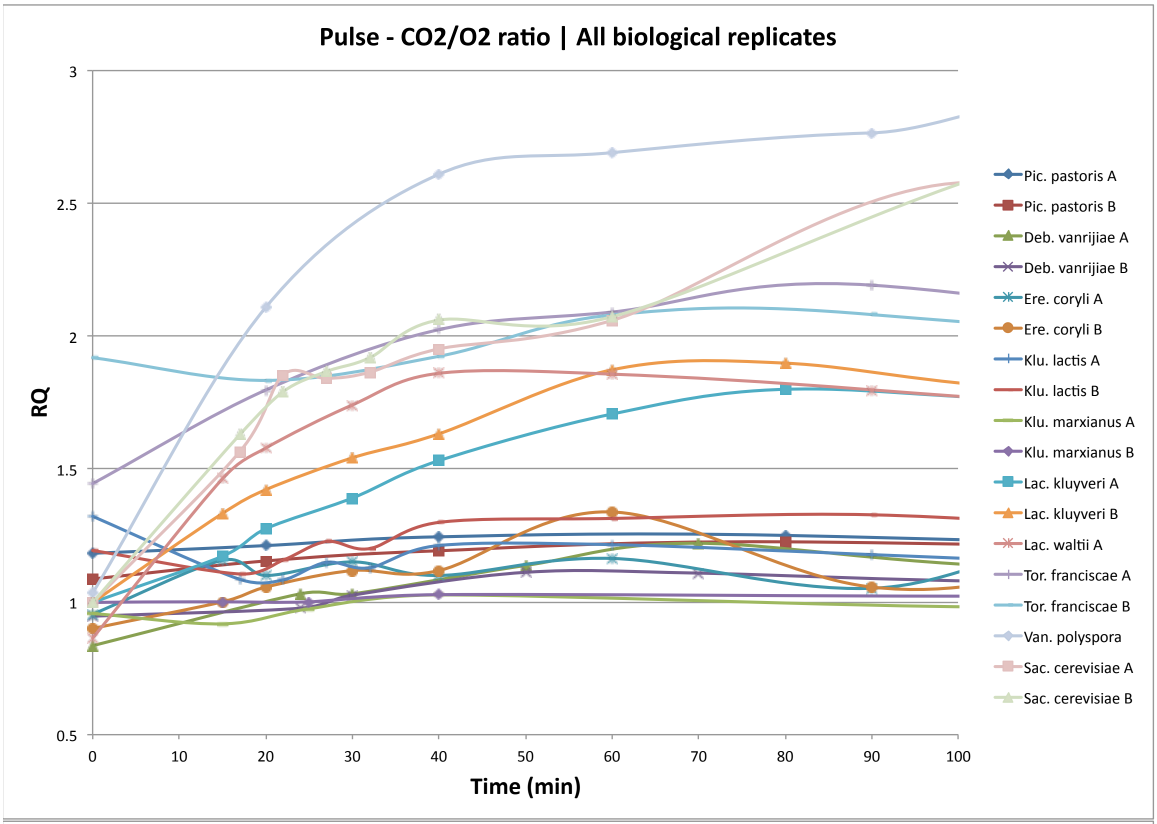


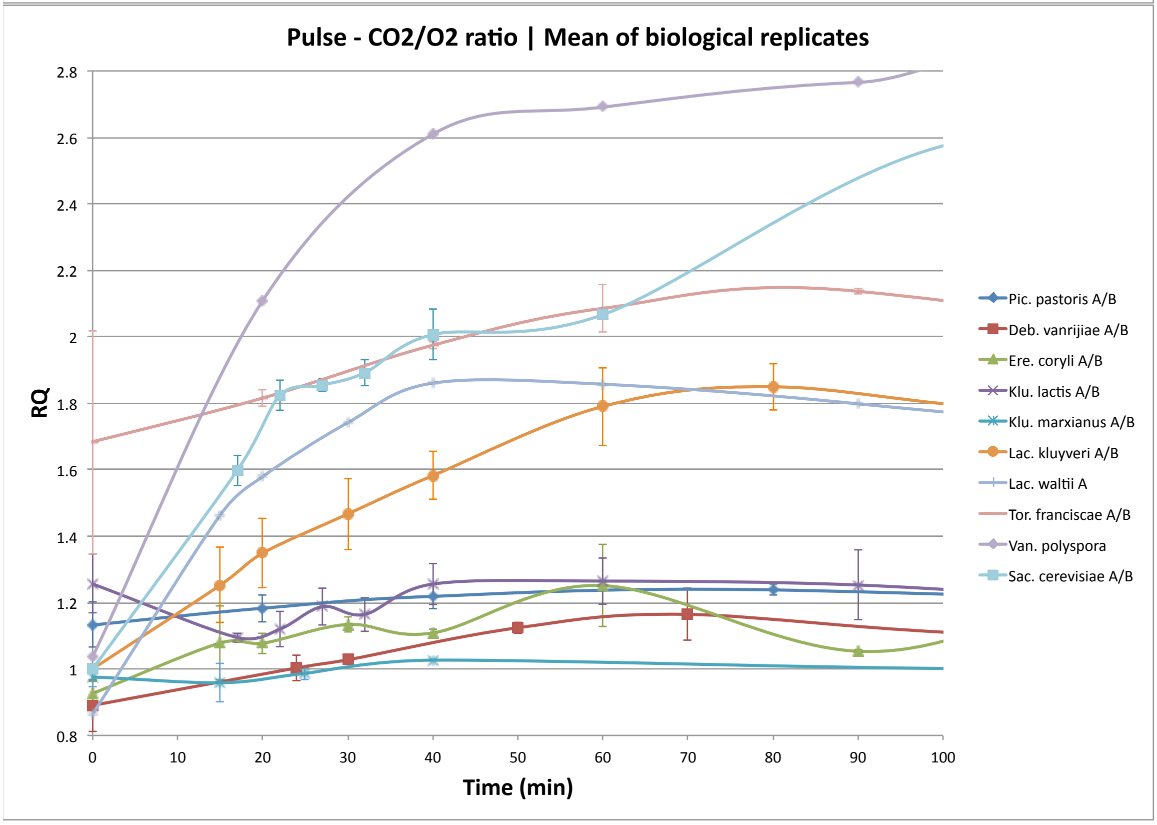


**Figure S6: Respiratory quotient (first 100 min).** Yeast species were investigated for their carbon dioxide production rates and oxygen consumption rates. The ratio of these variables ($CO_2/O_2$) defines the RQ, which can be used to quantify fermentation that occurs at values greater than 1. This figure illustrates the RQ at all time points and for all investigated species, which are not shown in figure 4. The average RQ of two replicates for each time point with error bars corresponding to the standard deviation are shown.

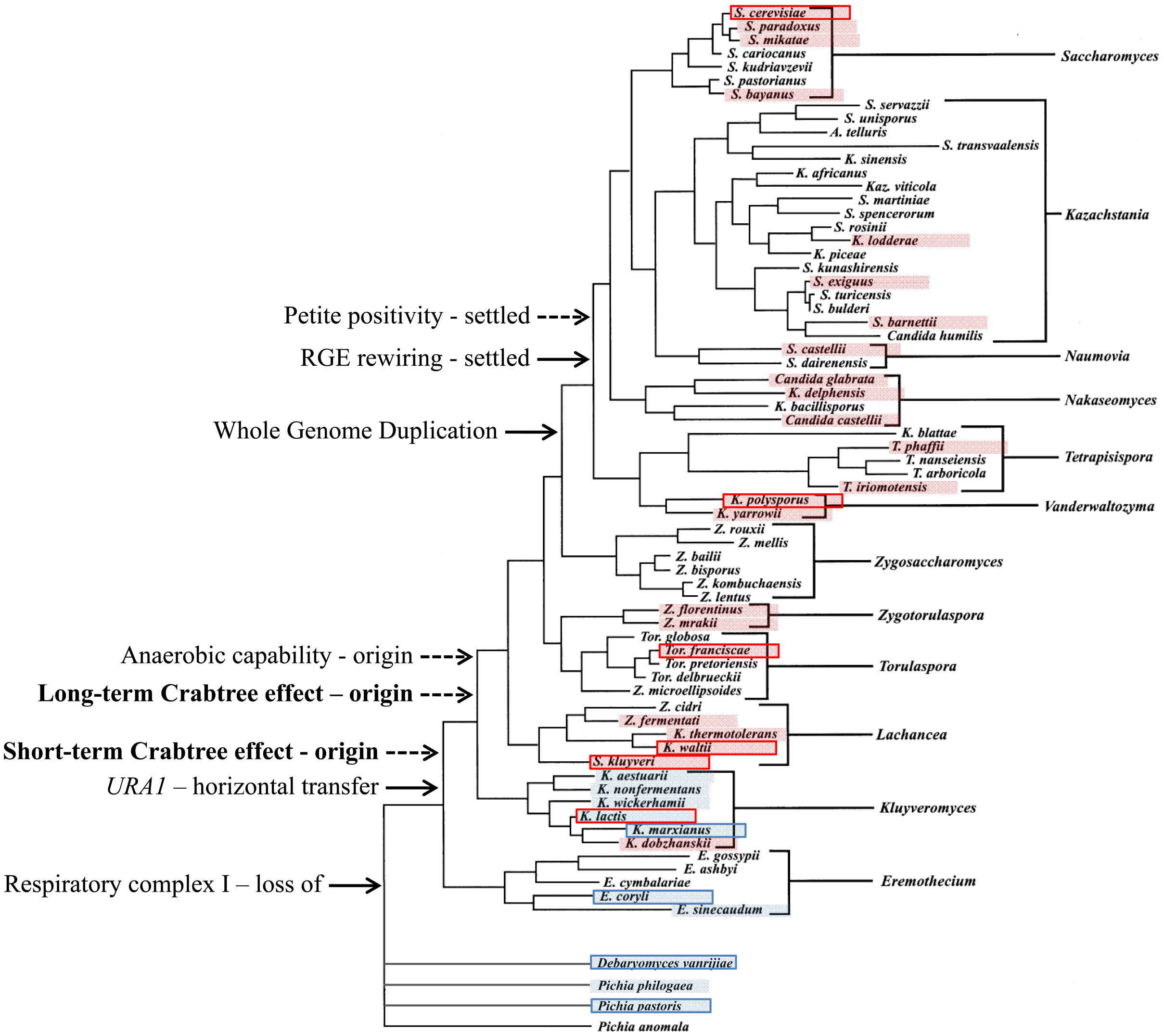


**Figure S7: Yeast carbon metabolism and phylogeny.** This figure illustrate the distribution of short-term Crabtree effect and long-term Crabtree effect as deduced from this study and a previous study [2]. Yeast species that are framed in red are short-term Crabtree positive and the ones that are framed in blue are short-term Crabtree negative. Similarly, yeast species that are highlighted in red are long-term Crabtree positive and the ones that are highlighted in blue are long-term Crabtree negative. Several evolutionary events that are relevant for the modern traits are also shown. Some events that have left a clear fingerprint in the modern genomes (whole arrows), such as the rewiring of RGE (rapid growth elements) [3], the whole genome duplication event [4], the horizontal transfer of *URA1* [5], and the loss of respiratory complex I [6] have been more precisely timed, while the timing of complex traits (broken arrows), such as petite positivity and the capability of anaerobic growth [7], might be less precise. Figure reproduced from [8].

**Table S1: Yeast short-term Crabtree effect – ethanol, glucose and DW for all time points.** Steady-state (SS) cultures were provided with a pulse of excess glucose, and several parameters such as consumption rates of glucose, and production rates of ethanol and biomass (DW) were determined during time points.

| Species | Time (minutes) | Cons. rate*: Glucose | Prod. rate*: Ethanol | Growth rate*: DW |
|---|---|---|---|---|
| *Sac. cerevisiae B* | SS | 6.7 | 0.0 | 3.8 |
| | 5 - 20 | 16.9 | 5.4 | 2.3 |
| | 20 - 40 | 17.8 | 8.0 | 9.6 |
| | 40 - 60 | 33.2 | 5.9 | 0.6 |
| | 60 - 105 | 36.1 | 13.7 | 8.9 |
| *Sac. cerevisiae A* | SS | 6.3 | 0.0 | 3.8 |
| | 5 - 20 | 21.9 | 5.3 | 1.4 |
| | 20 - 40 | 25.3 | 6.5 | -2.0 |
| | 40 - 60 | 29.5 | 12.0 | 14.5 |
| | 60 - 105 | 35.0 | 12.8 | 8.1 |
| *Van. polysporus* | SS | 6.8 | 0.0 | 3.8 |
| | 5 - 20 | 46.4 | 8.2 | 3.7 |
| | 20 - 40 | 31.7 | 16.6 | 10.6 |
| | 40 - 60 | 40.5 | 15.8 | 5.0 |
| | 60 - 90 | 37.6 | 17.1 | 10.1 |
| | 90 - 130 | 37.1 | -3.8 | 5.4 |
| *Tor. franciscae B* | SS | 6.0 | 0.0 | 3.8 |
| | 5 - 20 | 11.4 | 3.0 | 8.5 |
| | 20 - 40 | 13.1 | 3.6 | 2.3 |
| | 40 - 60 | 12.4 | 3.8 | 1.5 |
| | 60 - 90 | 14.9 | 5.5 | 3.9 |
| | 90 - 150 | 33.7 | 8.2 | 4.5 |
| *Tor. franciscae A* | SS | 5.6 | 0.0 | 3.8 |
| | 5 - 20 | 11.9 | 2.6 | 5.0 |
| | 20 - 40 | 13.2 | 2.7 | 2.9 |
| | 40 - 60 | 13.1 | 3.8 | -1.5 |
| | 60 - 90 | 15.5 | 5.4 | 5.7 |
| | 90 - 150 | 31.4 | 8.5 | 2.4 |
| *Lac. waltii B* | SS | 5.9 | 0.0 | 3.8 |
| | 5 - 20 | 22.3 | 5.1 | 14.6 |
| | 20 - 40 | 19.6 | 4.1 | -0.7 |
| | 40 - 60 | 20.4 | 5.2 | 7.5 |
| | 60 - 90 | 16.7 | 4.7 | 6.7 |
| | 90 - 135 | 18.2 | 4.7 | 5.4 |
| *Lac. waltii A* | SS | 5.7 | 0.0 | 3.8 |
| | 5 - 20 | 18.7 | 4.5 | 11.2 |
| | 20 - 40 | 19.9 | 4.4 | 0.7 |
| | 40 - 60 | 18.5 | 4.4 | 3.3 |
| | 60 - 90 | 18.5 | 4.9 | 6.3 |
| | 90 - 135 | 18.5 | 4.8 | 6.3 |
| *Lac. kluyverii B* | SS | 6.9 | 0.0 | 3.8 |
| | 5 - 20 | 16.0 | 3.2 | -4.5 |
| | 20 - 40 | 17.5 | 6.4 | 1.4 |
| | 40 - 60 | 13.6 | 6.5 | 7.2 |
| | 60 - 80 | 24.5 | 7.3 | 1.9 |
| | 80 - 110 | 24.8 | 8.9 | 6.4 |
| | 110 - 170 | 25.9 | 10.9 | 13.9 |
| *Lac. kluyverii A* | SS | 7.1 | 0.0 | 3.8 |

| | | | | |
|---|---|---|---|---|
| | 5 - 20 | 20.2 | 2.7 | 1.8 |
| | 20 - 40 | 14.0 | 7.4 | 0.0 |
| | 40 - 60 | 21.5 | 10.1 | 2.0 |
| | 60 - 80 | 30.8 | 4.3 | 6.4 |
| | 80 - 110 | 18.5 | 11.5 | 8.9 |
| | 110 - 170 | 26.2 | 9.8 | 8.4 |
| *Klu. lactis B* | SS | 6.5 | 0.0 | 3.8 |
| | 5 - 20 | 14.6 | 0.1 | 6.9 |
| | 20 - 40 | 22.2 | 2.1 | 13.3 |
| | 40 - 60 | 17.8 | 4.7 | 3.9 |
| | 60 - 90 | 17.3 | 3.3 | 7.0 |
| | 90 - 120 | 15.9 | 2.3 | 7.3 |
| | 120 - 150 | 18.0 | 1.8 | 5.3 |
| | 150 - 210 | 16.3 | 0.2 | 5.5 |
| *Klu. lactis A* | SS | 6.4 | 0.0 | 3.8 |
| | 5 - 20 | 17.3 | 1.6 | 6.4 |
| | 20 - 40 | 17.7 | 2.3 | 8.8 |
| | 40 - 60 | 18.6 | 3.2 | 5.4 |
| | 60 - 90 | 16.9 | 3.9 | 7.6 |
| | 90 - 120 | 14.3 | 2.6 | 5.8 |
| | 120 - 150 | 19.5 | 3.4 | 4.0 |
| | 150 - 210 | 15.2 | 0.0 | 8.7 |
| *Klu. marxianus B* | SS | 6.1 | 0.0 | 3.8 |
| | 5 - 20 | 14.8 | -1.1 | 8.1 |
| | 20 - 40 | 10.6 | -1.1 | 11.4 |
| | 40 - 60 | 15.0 | 0.0 | 7.0 |
| | 60 - 105 | 13.5 | 0.0 | 8.2 |
| | 105 - 150 | 12.6 | 0.1 | 8.4 |
| | 150 - 210 | 11.7 | 0.0 | 7.7 |
| *Klu. marxianus A* | SS | 6.0 | 0.0 | 3.8 |
| | 5 - 20 | 14.4 | 0.9 | 9.8 |
| | 20 - 40 | 15.4 | 0.0 | 9.3 |
| | 40 - 60 | 10.8 | 0.0 | 8.5 |
| | 60 - 105 | 13.5 | 0.0 | 8.7 |
| | 105 - 150 | 12.0 | 0.1 | 9.0 |
| | 150 - 210 | 11.8 | 0.0 | 7.1 |
| *Ere. coryli B* | SS | 4.3 | 0.0 | 3.8 |
| | 5 - 20 | 1.2 | 0.4 | 4.4 |
| | 20 - 40 | 3.0 | 0.0 | 2.0 |
| | 40 - 60 | 4.1 | 0.4 | 1.2 |
| | 60 - 90 | 3.0 | -0.1 | 1.3 |
| | 90 - 120 | 2.9 | -0.2 | 1.5 |
| | 120 - 180 | 2.6 | -0.1 | 1.4 |
| | 180 - 240 | 2.2 | 0.0 | 0.4 |
| | 240 - 330 | 2.3 | 0.0 | 1.9 |
| | 330 - 495 | 2.1 | 0.1 | 1.3 |
| | 495 - 572 | 1.4 | -0.1 | 0.2 |
| | 572 - 735 | 1.6 | 0.0 | 0.7 |
| *Ere. coryli A* | SS | 3.9 | 0.0 | 3.8 |
| | 5 - 20 | 4.3 | 0.7 | 5.3 |
| | 20 - 40 | 5.1 | 0.1 | 1.2 |
| | 40 - 60 | 3.4 | 1.1 | 3.8 |
| | 60 - 90 | 2.6 | -1.1 | 1.5 |
| | 90 - 120 | 2.9 | 0.0 | 2.4 |
| | 120 - 180 | 3.0 | 0.0 | 1.0 |

| | | | | |
|---|---|---|---|---|
| | 180 - 240 | 2.0 | 0.0 | 2.3 |
| | 240 - 330 | 2.4 | 0.0 | 1.1 |
| | 330 - 495 | 2.4 | 0.1 | 1.1 |
| | 495 - 572 | 0.0 | 8.5 | -0.3 |
| | 572 - 735 | 0.0 | 8.9 | -0.1 |
| *Deb. vanrijiae B* | SS | 5.5 | 0.0 | 3.8 |
| | 5 - 25 | 9.7 | 0.0 | 1.7 |
| | 25 - 50 | 5.2 | 0.0 | 6.4 |
| | 50 - 70 | 5.2 | 0.0 | 6.8 |
| | 70 - 110 | 5.6 | 0.0 | 3.2 |
| | 110 - 200 | 7.2 | 0.0 | 4.8 |
| | 200 - 260 | 15.2 | 0.0 | 9.9 |
| *Deb. vanrijiae A* | SS | 5.5 | 0.0 | 3.8 |
| | 5 - 25 | 7.2 | -0.4 | 4.0 |
| | 25 - 50 | 5.6 | 0.0 | 5.9 |
| | 50 - 70 | 5.7 | -0.1 | 4.4 |
| | 70 - 110 | 6.7 | 0.0 | 4.8 |
| | 110 - 200 | 6.7 | 0.0 | 4.7 |
| | 200 - 260 | 14.6 | 0.0 | 10.0 |
| *Pic. pastoris B* | SS | 5.2 | 0.0 | 3.8 |
| | 5 - 20 | 14.2 | 0.0 | 13.9 |
| | 20 - 40 | 10.8 | 0.0 | 4.9 |
| | 40 - 80 | 8.9 | 0.0 | 11.4 |
| | 80 - 230 | 7.4 | 0.0 | 3.5 |
| *Pic. pastoris A* | SS | 5.1 | 0.0 | 3.8 |
| | 5 - 20 | 17.3 | 0.0 | 20.2 |
| | 20 - 40 | 9.0 | 0.0 | 5.5 |
| | 40 - 80 | 7.9 | 0.0 | 6.4 |
| | 80 - 230 | 7.5 | 0.0 | 3.7 |

* C-mmol/gDW*h

**Table S2: Yeast short-term Crabtree effect – $O_2$, $CO_2$ and RQ for all time points.** Steady-state (SS) cultures were provided with a pulse of glucose, and several parameters such as consumption rates of $O_2$, production rates of $CO_2$, and respiratory quotient were monitored at different time points.

| Species | Time (minutes) | Prod. rate*: CO2 | Cons. rate*: O2 | RQ |
|---|---|---|---|---|
| *Sac. cerevisiae B* | SS | 2.8 | 2.8 | 1.0 |
| | 20 | 5.4 | 3.4 | 1.6 |
| | 40 | 6.9 | 3.6 | 1.9 |
| | 60 | 7.4 | 3.6 | 2.1 |
| | 105 | 8.6 | 3.7 | 2.3 |
| *Sac. cerevisiae A* | SS | 2.6 | 2.6 | 1.0 |
| | 20 | 5.6 | 3.0 | 1.9 |
| | 40 | 7.5 | 3.9 | 1.9 |
| | 60 | 7.9 | 3.9 | 2.0 |
| | 105 | 8.8 | 3.8 | 2.3 |
| *Van. polysporus* | SS | 2.8 | 2.7 | 1.0 |
| | 20 | 7.0 | 4.0 | 1.7 |
| | 40 | 9.3 | 4.0 | 2.3 |
| | 60 | 9.9 | 3.7 | 2.7 |
| | 90 | 9.8 | 3.6 | 2.7 |
| | 130 | 9.5 | 3.4 | 2.8 |
| *Tor. franciscae B* | SS | 2.3 | 1.2 | 1.9 |
| | 20 | 3.3 | 1.7 | 1.9 |
| | 40 | 4.7 | 2.5 | 1.9 |
| | 60 | 5.3 | 2.6 | 2.0 |
| | 90 | 5.9 | 2.9 | 2.1 |
| | 150 | 6.3 | 3.2 | 1.9 |
| *Tor. franciscae A* | SS | 2.1 | 1.5 | 1.4 |
| | 20 | 3.1 | 2.0 | 1.5 |
| | 40 | 4.4 | 2.3 | 1.9 |
| | 60 | 5.0 | 2.4 | 2.1 |
| | 90 | 5.6 | 2.6 | 2.1 |
| | 150 | 6.0 | 3.0 | 2.0 |
| *Lac. waltii B* | SS | Nd | Nd | Nd |
| | 20 | Nd | Nd | Nd |
| | 40 | Nd | Nd | Nd |
| | 60 | Nd | Nd | Nd |
| | 90 | Nd | Nd | Nd |
| | 135 | Nd | Nd | Nd |
| *Lac. waltii A* | SS | 2.6 | 3.0 | 0.9 |
| | 20 | 4.0 | 3.6 | 1.1 |
| | 40 | 5.8 | 3.4 | 1.7 |
| | 60 | 6.0 | 3.3 | 1.9 |
| | 90 | 5.9 | 3.3 | 1.8 |
| | 135 | 5.8 | 3.3 | 1.7 |
| *Lac. kluyverii B* | SS | 2.9 | 2.9 | 1.0 |
| | 20 | 4.4 | 3.6 | 1.2 |
| | 40 | 6.2 | 4.1 | 1.5 |
| | 60 | 7.1 | 4.1 | 1.8 |
| | 80 | 7.6 | 4.1 | 1.9 |
| | 110 | 7.9 | 4.3 | 1.8 |
| | 170 | 7.2 | 4.1 | 1.8 |
| *Lac. kluyverii A* | SS | 3.2 | 3.2 | 1.0 |

| | | | | |
|---|---|---|---|---|
| | 20 | 4.5 | 3.9 | 1.2 |
| | 40 | 6.3 | 4.5 | 1.4 |
| | 60 | 7.4 | 4.6 | 1.6 |
| | 80 | 8.0 | 4.6 | 1.8 |
| | 110 | 8.1 | 4.5 | 1.8 |
| | 170 | 7.8 | 4.6 | 1.7 |
| *Klu. lactis B* | SS | 0.9 | 0.9 | 1.0 |
| | 20 | 4.7 | 3.6 | 1.3 |
| | 40 | 5.4 | 4.4 | 1.2 |
| | 60 | 5.7 | 4.3 | 1.3 |
| | 90 | 5.8 | 4.4 | 1.3 |
| | 120 | 5.8 | 4.4 | 1.3 |
| | 150 | 5.7 | 4.7 | 1.2 |
| | 210 | 5.6 | 5.3 | 1.1 |
| *Klu. lactis A* | SS | 0.9 | 1.0 | 1.0 |
| | 20 | 4.5 | 3.6 | 1.3 |
| | 40 | 5.0 | 4.3 | 1.1 |
| | 60 | 5.1 | 4.2 | 1.2 |
| | 90 | 5.1 | 4.3 | 1.2 |
| | 120 | 5.1 | 4.4 | 1.2 |
| | 150 | 5.1 | 4.6 | 1.1 |
| | 210 | 4.8 | 4.8 | 1.0 |
| *Klu. marxianus B* | SS | 2.5 | 2.1 | 1.2 |
| | 20 | 1.5 | 1.5 | 1.0 |
| | 40 | 1.5 | 1.4 | 1.0 |
| | 60 | 1.5 | 1.4 | 1.0 |
| | 105 | 1.5 | 1.4 | 1.0 |
| | 150 | 1.4 | 1.4 | 1.0 |
| | 210 | 1.4 | 1.3 | 1.1 |
| *Klu. marxianus A* | SS | 3.0 | 2.2 | 1.3 |
| | 20 | 1.5 | 1.6 | 1.0 |
| | 40 | 1.5 | 1.5 | 1.0 |
| | 60 | 1.5 | 1.5 | 1.0 |
| | 105 | 1.5 | 1.5 | 1.0 |
| | 150 | 1.5 | 1.5 | 1.0 |
| | 210 | 1.4 | 1.4 | 1.0 |
| *Ere. coryli B* | SS | 0.7 | 0.8 | 0.9 |
| | 20 | 0.7 | 0.8 | 1.0 |
| | 40 | 0.7 | 0.7 | 1.1 |
| | 60 | 0.7 | 0.6 | 1.2 |
| | 90 | 0.7 | 0.6 | 1.2 |
| | 120 | 0.7 | 0.6 | 1.1 |
| | 180 | 0.6 | 0.5 | 1.2 |
| | 240 | 0.6 | 0.5 | 1.2 |
| | 330 | 0.6 | 0.5 | 1.1 |
| | 495 | 0.5 | 0.5 | 1.1 |
| | 572 | 0.5 | 0.5 | 1.0 |
| | 735 | 0.4 | 0.4 | 0.9 |
| *Ere. coryli A* | SS | 0.7 | 0.8 | 1.0 |
| | 20 | 0.8 | 0.8 | 1.1 |
| | 40 | 0.8 | 0.7 | 1.1 |
| | 60 | 0.8 | 0.7 | 1.1 |
| | 90 | 0.7 | 0.7 | 1.1 |
| | 120 | 0.7 | 0.6 | 1.1 |
| | 180 | 0.7 | 0.6 | 1.2 |

| | | | | |
|---|---|---|---|---|
| | 240 | 0.6 | 0.5 | 1.2 |
| | 330 | 0.6 | 0.5 | 1.1 |
| | 495 | 0.6 | 0.5 | 1.1 |
| | 572 | 0.5 | 0.5 | 1.0 |
| | 735 | 0.4 | 0.5 | 0.9 |
| *Deb. vanrijiae B* | SS | 1.9 | 2.0 | 0.9 |
| | 25 | 2.1 | 2.2 | 1.0 |
| | 50 | 2.1 | 2.1 | 1.0 |
| | 70 | 2.0 | 1.8 | 1.1 |
| | 110 | 2.0 | 1.8 | 1.1 |
| | 200 | 2.2 | 2.1 | 1.1 |
| | 260 | 2.6 | 2.5 | 1.1 |
| *Deb. vanrijiae A* | SS | 1.7 | 2.1 | 0.8 |
| | 25 | 2.0 | 2.2 | 0.9 |
| | 50 | 2.2 | 2.1 | 1.1 |
| | 70 | 2.1 | 1.8 | 1.2 |
| | 110 | 2.0 | 1.7 | 1.2 |
| | 200 | 2.2 | 2.0 | 1.1 |
| | 260 | 2.6 | 2.4 | 1.1 |
| *Pic. pastoris B* | SS | 1.5 | 1.4 | 1.1 |
| | 20 | 2.1 | 1.9 | 1.1 |
| | 40 | 2.3 | 2.0 | 1.2 |
| | 80 | 2.1 | 1.7 | 1.2 |
| | 230 | 2.0 | 1.7 | 1.2 |
| *Pic. pastoris A* | SS | 1.5 | 1.3 | 1.2 |
| | 20 | 2.0 | 1.8 | 1.1 |
| | 40 | 2.4 | 1.9 | 1.2 |
| | 80 | 2.2 | 1.7 | 1.2 |
| | 230 | 2.3 | 1.9 | 1.2 |

** mmol/gDW*h
Nd = Not determined

**Table S3: Homogeneity test of variance between two metabolic groups on investigated growth parameters.** Bartlett's K-squared and Levene's statistical tests reveal consistent results. Both tests could detect significant (at alpha =5%) homogeneity of variance between SCN and SCP yeasts (including K. lactis) for ethanol production rate and RQ, but fail to reveal any significant level of homoscedasticity among groups for all other investigated parameters.

| Statistical analysis | p-value | df | K-sq/F-val |
|---|---|---|---|
| Bartlett's K-squared: Glucose cons. rate | 1.78E-01 | 1 | 1.81 |
| Bartlett's K-squared: Ethanol prod. rate | 2.75E-03 | 1 | 8.97 |
| Bartlett's K-squared: O2 cons. rate | 4.47E-01 | 1 | 0.58 |
| Bartlett's K-squared: CO2 prod. rate | 7.28E-02 | 1 | 3.22 |
| Bartlett's K-squared: RQ | 1.25E-03 | 1 | 10.42 |
| Levene's test: Glucose cons. rate | 8.62E-01 | 17 | 0.03 |
| Levene's test: Ethanol prod. rate | 3.51E-02 | 17 | 5.24 |
| Levene's test: O2 cons. rate | 8.70E-01 | 16 | 0.03 |
| Levene's test: CO2 prod. rate | 2.86E-01 | 16 | 1.22 |
| Levene's test: RQ | 1.54E-03 | 16 | 14.52 |

**Table S4: Statistical tests among three metabolic groups on investigated growth parameters.** Tests on two parameters (under x- and y-column) are considered to be significant at a significance level of α = 5% or at a P-value lower than 0.05. Yeast species were grouped in the following way. Group 1 comprises SCN yeasts (but not *K. lactis*), Group 2 contains only *K. lactis*, and Group 3 comprises all SCP yeasts (but not *K. lactis*). Kruskal-Wallis test amongst three groups reveal significant differences in all parameters, while ANOVA test failed to reveal any significant differences in glucose consumption rate amongst groups. Hence, at least two groups are not significantly different on glucose consumption rate. When the Tukey-Kramer test was performed on all combinations of groups, a significant difference between group 1 and 3 on all parameters except for glucose consumption rate could be detected. The same test could also detect significant differences between group 1 and 2 only on $O_2$ consumption and $CO_2$ production rate. Finally, a significant difference between group 1 and 3 could be detected only on ethanol production rate. Thus, our result suggests that *K. lactis* can be considered as intermediate SCN and SCP.

| **Statistical analysis** | **Par. X** | **Par. Y** | **α = 5%** | **P-value** | **df** | **t-val** | **R2** |
|---|---|---|---|---|---|---|---|
| ANOVA | Groups | Glucose cons. rate | No | 6.7E-02 | 16 | 3.2 | 0.29 |
| | | Ethanol prod. rate | Yes | 2.1E-05 | 16 | 22.8 | 0.74 |
| | | O2 cons. rate | | 5.8E-04 | 15 | 12.7 | 0.63 |
| | | CO2 prod. rate | | 3.0E-05 | 15 | 22.6 | 0.75 |
| | | RQ | | 1.6E-03 | 15 | 10.1 | 0.57 |
| Kruskal-Wallis test | Groups | Glucose cons. rate | Yes | 3.3E-02 | 2 | 6.8 | - |
| | | Ethanol prod. rate | | 9.1E-04 | 2 | 14.0 | - |
| | | O2 cons. rate | | 8.1E-03 | 2 | 9.6 | - |
| | | CO2 prod. rate | | 1.7E-03 | 2 | 12.7 | - |
| | | RQ | | 1.7E-03 | 2 | 12.7 | - |
| Tukey-Kramer: Glucose cons. rate | Group 1 | Group 2 | No | 6.8E-01 | - | - | - |
| | | Group 3 | | 5.4E-02 | - | - | - |
| | Group 2 | Group 3 | | 7.5E-01 | - | - | - |
| Tukey-Kramer: Ethanol prod. rate | Group 1 | Group 2 | No | 7.4E-01 | - | - | - |
| | | Group 3 | Yes | 1.8E-05 | - | - | - |
| | Group 2 | Group 3 | | 1.1E-02 | - | - | - |
| Tukey-Kramer: O2 cons. rate | Group 1 | Group 2 | Yes | 6.4E-03 | - | - | - |
| | | Group 3 | | 1.2E-03 | - | - | - |
| | Group 2 | Group 3 | No | 7.0E-01 | - | - | - |
| Tukey-Kramer: CO2 prod. rate | Group 1 | Group 2 | Yes | 3.4E-03 | - | - | - |
| | | Group 3 | | 3.3E-05 | - | - | - |
| | Group 2 | Group 3 | No | 9.9E-01 | - | - | - |
| Tukey-Kramer: RQ | Group 1 | Group 2 | No | 3.2E-01 | - | - | - |
| | | Group 3 | Yes | 1.2E-03 | - | - | - |
| | Group 2 | Group 3 | No | 3.9E-01 | - | - | - |

# References


1. Kiers, J., Zeeman, A. M., Luttik, M., Thiele, C., Castrillo, J. I., Steensma, H. Y., van Dijken, J. P. & Pronk, J. T. (1998) Regulation of alcoholic fermentation in batch and chemostat cultures of *Kluyveromyces lactis* CBS 2359, *Yeast.* **14**, 459-69.
2. Hagman, A., Sall, T., Compagno, C. & Piskur, J. (2013) Yeast "make-accumulate-consume" life strategy evolved as a multi-step process that predates the whole genome duplication, *PLoS One.* **8**, e68734.
3. Ihmels, J., Bergmann, S., Gerami-Nejad, M., Yanai, I., McClellan, M., Berman, J. & Barkai, N. (2005) Rewiring of the yeast transcriptional network through the evolution of motif usage, *Science.* **309**, 938-40.
4. Wolfe, K. H. & Shields, D. C. (1997) Molecular evidence for an ancient duplication of the entire yeast genome, *Nature.* **387**, 708-13.
5. Gojkovic, Z., Knecht, W., Zameitat, E., Warneboldt, J., Coutelis, J. B., Pynyaha, Y., Neuveglise, C., Moller, K., Loffler, M. & Piskur, J. (2004) Horizontal gene transfer promoted evolution of the ability to propagate under anaerobic conditions in yeasts, *Molecular Genetics and Genomics : MGG.* **271**, 387-93.
6. Dujon, B. (2010) Yeast evolutionary genomics, *Nature reviews Genetics.* **11**, 512-24.
7. Merico, A., Sulo, P., Piskur, J. & Compagno, C. (2007) Fermentative lifestyle in yeasts belonging to the *Saccharomyces* complex, *The FEBS journal.* **274**, 976-89.
8. Kurtzman, C. P. & Robnett, C. J. (2003) Phylogenetic relationships among yeasts of the '*Saccharomyces* complex' determined from multigene sequence analyses, *FEMS Yeast Res.* **3**, 417-32.
